# Overexpression of *VqWRKY31* enhances powdery mildew resistance in grapevine by promoting salicylic acid signaling and specific metabolite synthesis

**DOI:** 10.1093/hr/uhab064

**Published:** 2022-01-19

**Authors:** Wuchen Yin, Xianhang Wang, Hui Liu, Ya Wang, Steve van Nocker, Mingxing Tu, Jinghao Fang, Junqiang Guo, Zhi Li, Xiping Wang

**Affiliations:** 1 State Key Laboratory of Crop Stress Biology in Arid Areas, College of Horticulture, Northwest A&F University, Yangling, Shaanxi 712100, China; 2 Key Laboratory of Horticultural Plant Biology and Germplasm Innovation in Northwest China, Ministry of Agriculture, Northwest A&F University, Yangling, Shaanxi 712100, China; 3 College of Enology, Northwest A&F University, Yangling, Shaanxi 712100, China; 4 Department of Horticulture, Michigan State University, East Lansing, MI 48824, USA

## Abstract

Powdery mildew (PM), caused by the fungal pathogen *Erysiphe necator*, is one of the most destructive diseases of grapevine (*Vitis vinifera* and other *Vitis* spp.). Resistance to PM is an important goal for cultivar improvement, and understanding the underlying molecular mechanisms conditioning resistance is critical. Here, we report that transgenic expression of the WRKY transcription factor gene *VqWRKY31* from the PM-resistant species *Vitis quinquangularis* conferred resistance to PM in *V. vinifera* through promoting salicylic acid signaling and specific metabolite synthesis. *VqWRKY31* belongs to the WRKY IIb subfamily, and expression of the *VqWRKY31* gene was induced in response to *E. necator* inoculation. Transgenic *V. vinifera* plants expressing *VqWRKY31* were substantially less susceptible to *E. necator* infection, and this was associated with increased levels of salicylic acid and reactive oxygen species. Correlation analysis of transcriptomic and metabolomic data revealed that *VqWRKY31* promoted expression of genes in metabolic pathways and the accumulation of many disease resistance-related metabolites, including stilbenes, flavonoids, and proanthocyanidins. In addition, results indicated that *VqWRKY31* can directly bind to the promoters of two structural genes in stilbene synthesis, *STS9* and *STS48,* and activate their expression. Based on our results, we propose a model where *VqWRKY31* enhances grapevine PM resistance through activation of salicylic acid defense signaling and promotion of specific disease resistance-related metabolite synthesis. These findings can be directly exploited for molecular breeding strategies to produce PM-resistant grapevine germplasm.

## Introduction

Grapes are among the most ancient and ubiquitously cultivated fruits worldwide. However, the sustainability of grape-related industries is increasingly threatened by several diseases. Among these, powdery mildew (PM), which is caused by the biotrophic fungus *Erysiphe necator* Schw. (syn. *Uncinula necator*), is arguably the most serious [1]. Although PM can be partially controlled through the use of fungicides, this practice can negatively affect the environment, is costly and time-consuming for producers, and is not sustainable [2]. Therefore, genetic resistance to PM has become a top breeding goal for grapevine cultivar improvement, and to implement this, it is critical to identify resistance-related genes and molecular pathways in grapevine.

Currently, worldwide grape production mainly utilizes European grapevine (*Vitis vinifera* L.) cultivars, which generally are highly susceptible to PM. However, many wild grapevines from North America and eastern Asia show strong resistance to PM [3, 4]. Many efforts have been made to introgress PM resistance from these wild species into *V. vinifera*, and some interspecific hybrids with PM resistance have been produced [5]. In addition, several genetic loci influencing PM resistance have been identified, including *RUN1*, *RUN2*, *REN2*, *REN3*, *REN4*, *REN5*, *REN6*, and *REN7* [1]. Numerous studies of the molecular mechanisms of PM resistance in grapevine have also been initiated. Many transcription factor genes have been identified from wild grapevines that are induced in response to *E. necator* inoculation, including members of the WRKY, ERF, and MYB families [6–8], and at least some of these genes were shown to confer PM resistance when expressed in *Arabidopsis thaliana* (arabidopsis) [8, 9]. However, the mechanisms by which these genes are activated and regulate downstream gene expression generally remain unknown.

Plants growing in natural environments are continuously exposed to pathogens and have developed a sophisticated immune system. Salicylic acid (SA), an endogenous hormone, plays an important role in pathogen response and disease resistance by activating the SA immune signaling pathway [10]. SA signaling can facilitate the expression of pathogenesis-related (*PR*) genes and contribute to the hypersensitive response (HR) [10, 11]. The HR is a form of programmed cell death that results in necrosis of tissues at sites of attempted infection, and is critical for limiting the progression of infection [11, 12]. The SA defense signaling pathway is often accompanied by rapid generation of reactive oxygen species (ROS), which act synergistically with SA to drive the HR [11, 13].

In addition to SA-mediated defense signaling, some secondary metabolites are also vital contributors to plant disease resistance. For example, stilbenoids are a class of secondary metabolites that act as phytoalexins [14]. The synthesis of a typical stilbenoid compound, resveratrol, depends on stilbene synthase (STS). Overexpressing STS in grapevine or other plants has been shown to promote the accumulation of stilbenes and enhance disease resistance [15–17]. Resveratrol can be metabolized to form various other stilbene phytoalexins, such as ε-viniferin and pterostilbene, which can inhibit pathogen growth and contribute to the HR [18]. In addition to stilbenoids, flavonoids, such as the derivatives of naringenin, kaempferol, quercetin, and catechin, can directly inhibit microbial growth [19–22]. Proanthocyanidins, which are end products of flavonoid biosynthesis, are also widely involved in plant disease resistance and SA signaling [23, 24]. Flavonoids are synthesized through the action of several well-studied enzymes, including chalcone synthase (CHS), chalcone isomerase (CHI), flavonoid-3′,5′-hydroxylase (F3′5′H), flavanone 3-dioxygenase (FHT), flavonol synthase (FLS), and dihydroflavanol 4-reductase (DFR) [25, 26]. However, the mechanism by which flavonoid biosynthetic genes might be regulated in response to PM remains unclear.

Considerable research has shown that WRKY transcription factors also play an important role in plant immunity. In arabidopsis, the *WRKY46*, *WRKY70*, and *WRKY53* genes enhance immunity to *Pseudomonas syringae* [27], and *WRKY33* promotes resistance to necrotrophic *Botrytis cinerea* and *Alternaria brassicicola* [28]. The chrysanthemum *CmWRKY15* gene can be induced by SA treatment, and *CmWRKY15*-overexpressing transgenic lines showed increased resistance to *Puccinia horiana* [29]. Moreover, some WRKY genes from grapevine can confer enhanced pathogen resistance when expressed in arabidopsis [8, 9].

Here, we identified a WRKY transcription factor gene, *VqWRKY31*, from the PM-resistant wild grapevine *Vitis quinquangularis*, which was strongly induced in response to PM. We demonstrate that transgenic expression of *VqWRKY31* in *V. vinifera* can significantly inhibit the development of PM after inoculation with *E. necator*. Expression of *VqWRKY31* also activated SA defense signaling and altered the accumulation of stilbenes, flavonoids, and proanthocyanidins. We also show that *VqWRKY31* can directly regulate the expression of the stilbene synthesis genes *STS9* and *STS48*. Taken together, these results suggest that *VqWRKY31* is an important component in the regulation of grapevine resistance to PM, which may facilitate development of PM-resistant grapevine germplasm.

## Results

### Characterization of *VqWRKY31*

**Figure 1:**
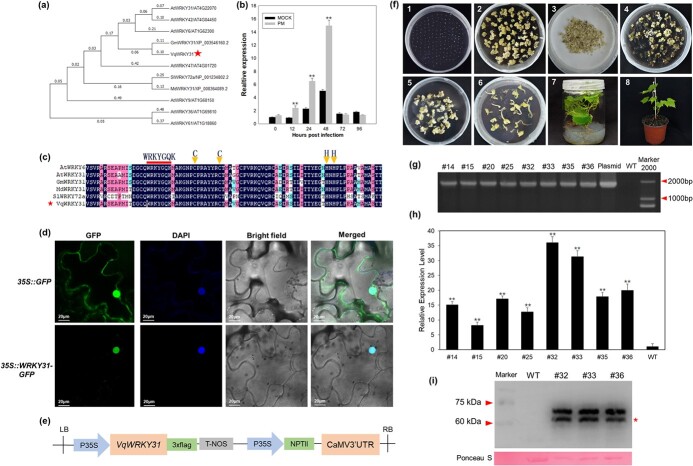
Characterization of *VqWRKY31*. **a** Phylogenetic analysis of VqWRKY31. Gm, *Glycine max*; Md, *Malus domestica*; Vq, *Vitis quinquangularis*; At, *Arabidopsis thaliana*; Sl, *Solanum lycopersicum*. The red asterisk indicates VqWRKY31. **b** Relative expression of *VqWRKY31* in leaves of *V. quinquangularis* after *E. necator* inoculation. The reference gene used was *ACTIN7* (XM_002282480). Data represent means ± standard errors (*n* = 3). Asterisks represent significant differences (^*^P < .05, ^**^P < .01, Student’s *t* test) between inoculated and mock-inoculated plants at the specified time point. **c** Multiple amino acid sequence alignments between VqWRKY31 and other related WRKY proteins. The red line indicates the WRKYGQK conserved domain sequence; the yellow triangles indicate the zinc-finger motifs. The red asterisk indicates VqWRKY31. **d** Subcellular localization of VqWRKY31 in tobacco leaves. Bar = 20 μm. **e** Schematic diagram of the CaMV35S-*VqWRKY31*–3 × Flag plasmid. **f***Agrobacterium*-mediated transformation of ‘Thompson Seedless’ (1, flower buds before induction; 2, pro-embryogenic masses induced from flower buds; 3, co-culture of pro-embryogenic masses and *Agrobacterium* GV3101; 4, 5, kanamycin-resistant embryogenic callus; 6, somatic embryos with kanamycin resistance; 7, transgenic plantlets grown in MS medium; 8, transgenic lines in a growth chamber. **g** Verification of T-DNA insertion in *VqWRKY31*-expressing lines. The numbers above represent the specific transgenic line. Plasmid, CaMV35S-*VqWRKY31*–3 × Flag plasmid; WT, wild type; Marker 2000, DNA Marker DS2000. **h** qRT–PCR analysis of *VqWRKY31* transcriptional level in *VqWRKY31*-expressing and control lines. The endogenous control used was *ACTIN7* (XM_002282480). Data represent means ± standard errors (*n* = 3). Asterisks represent significant differences (^**^P < .01, Student’s *t* test) between *VqWRKY31*-expressing and control lines. **i** Western blotting to detect expression of VqWRKY31-3 × Flag (~60.72 kDa) protein. Ponceau S staining served as the loading control. The red asterisk indicates the migration position expected for VqWRKY31-3 × Flag protein.

To investigate a potential role for *VqWRKY31* in the response to PM, we used quantitative real-time reverse transcription PCR (qRT–PCR) to evaluate the expression of *VqWRKY31* in the resistant *V. quinquangularis* accession ‘Shang-24’ after inoculation with *E. necator*. The results showed that the *VqWRKY31* transcript level was strongly increased from 12 to 48 hours after inoculation, with the expression peaking at 48 hours at a level 3.12 times higher than that of the mock-inoculated control (Fig. 1b).

We then cloned the *VqWRKY31* coding sequence (CDS) from ‘Shang-24’. A phylogenetic analysis indicated that *VqWRKY31* was most closely related to the arabidopsis subgroup IIb WRKYs, with highest homology to GmWRKY31, MdWRKY31, and SlWRKY72 (Fig. 1a). An alignment of the deduced peptide sequence of VqWRKY31 with these homologous proteins showed that the proteins contained a conserved WRKY DNA-binding domain and a C_2_H_2_ zinc finger motif, both of which are characteristic of group II WRKY members (Fig. 1c). We transformed the recombinant vector 35S-*VqWRKY31*-green fluorescent protein (GFP) and the non-modified 35S-GFP vector into tobacco leaves. In the epidermal cells of leaves transformed with the 35S-*VqWRKY31*-GFP recombinant vector, GFP fluorescence was present only in the nucleus, whereas in the epidermal cells of the control the GFP fluorescence signal was visualized in the nucleus and cytoplasm (Fig. 1d). This result suggested that VqWRKY31 is located in the nucleus.

As another approach to assess a potential function for *VqWRKY31* in PM resistance, we determined the effect of *Agrobacterium*-mediated transient overexpression of *VqWRKY31* on PM disease progression, using leaves of the PM-susceptible *V. vinifera* cultivar ‘Thompson Seedless’. The *VqWRKY31* CDS was engineered with a carboxyl (C)-terminal extension comprising three tandem repeats of the FLAG epitope, and expressed under control of the strong, constitutive 35S promoter. Leaves of ‘Thompson Seedless’ plants infiltrated with *Agrobacterium* containing the non-modified CaMV35S-3 × Flag empty vector were used as control. Expression of the *NPT II* gene was monitored to verify the presence of the vector, and this revealed vector-associated expression of *VqWRKY31* (Supplementary Fig. S1a Compared to the empty vector group, the mRNA transcripts of VqWRKY31 were significantly increased in the VqWRKY31-overexpressing group (Supplementary Fig. S1c)). We found that leaves infiltrated with the 35S-*VqWRKY31*-3 × Flag vector showed reduced PM disease symptoms, relative to the control, as early as 3 days post-inoculation (dpi) (Supplementary Fig. S1e). The hyphal length in control leaves was almost 4-fold longer than that in overexpressing leaves at 3 dpi (Supplementary Fig. S1d). Trypan blue staining also revealed many fewer hyphae and conidiophores in leaves infiltrated with the 35S-*VqWRKY31*-3 × Flag vector compared with control leaves (Supplementary Fig. S1b). These results indicated that *VqWRKY31* may play an important role in grapevine resistance against PM.

### Identification of transgenic *V. vinifera* lines expressing *VqWRKY31*

To further assess the role of *VqWRKY31* in enhancing disease resistance to PM, we generated 35S-*VqWRKY31*-3 × Flag *V. vinifera* transgenic plants, using *Agrobacterium*-mediated transformation of proembryonic masses derived from ‘Thompson Seedless’ (Fig. 1e and f; Supplementary Fig. S2). The presence of the transgene in the transformants was verified by PCR analysis (Fig. 1g), and the expression level of *VqWRKY31* was assessed by qRT–PCR (Fig. 1h). Three lines (#32, #33, and #36) with high *VqWRKY31* expression levels were selected for further analysis. Western blot analysis of these three lines indicated that the VqWRKY31-3 × Flag fusion protein was expressed as designed (Fig. 1i).

### *VqWRKY31* promotes resistance to PM in grapevine

**Figure 2:**
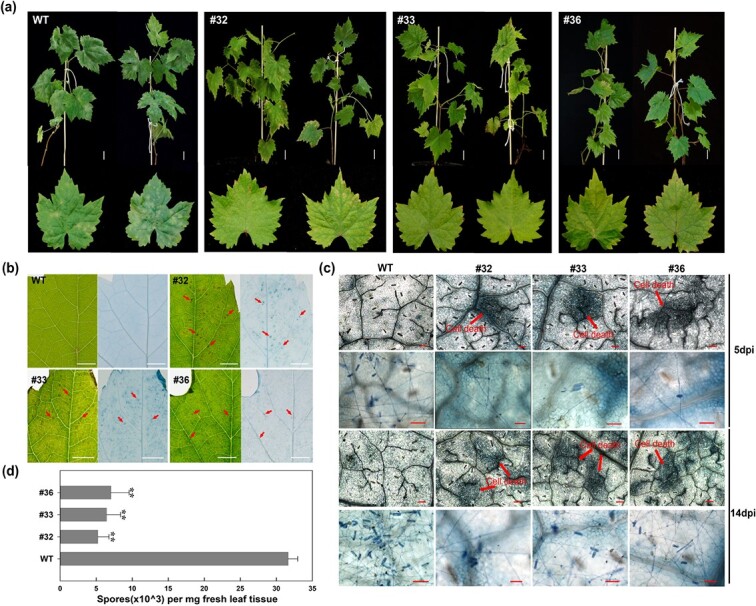
Transgenic expression of *VqWRKY31* in ‘Thompson Seedless’ enhances resistance to PM. **a** Phenotypes of *VqWRKY31*-expressing lines and non-transgenic control plants at 14 dpi (bar = 2.5 cm). **b** Representative leaves with backlighting at 3 dpi. Dark brown spots can be observed on leaves from *VqWRKY31*-expressing plants. Blue spots were apparent after staining with trypan blue. Arrows indicate spots (bar = 0.2 cm). **c** Phenotypes of leaves of *VqWRKY31*-expressing lines and control plants at 5 and 14 dpi stained with trypan blue. Arrows indicate foci of cell death. (bar = 50 μm). **d** Number of spores from 2 mg leaf tissue at 14 dpi. Data represent means ± standard errors (*n* = 3). Asterisks represent significant differences (^**^P < .01, Student’s *t* test).

To investigate whether the stable expression of *VqWRKY31* in ‘Thompson Seedless’ enhanced resistance to PM, we inoculated plants with *E. necator* conidia and observed the progression of disease symptoms over time. Non-transgenic ‘Thompson Seedless’ plants served as controls. At 3 dpi, leaves of non-transgenic control plants showed no obvious disease symptoms. In contrast, leaves of 35S-*VqWRKY31*-3 × Flag plants showed abundant dark brown spots, indicating that leaf cells may have undergone cell death (Fig. 2b). At 14 dpi, large whitish velvety layers, which is a typical presentation of PM, were seen on leaves of control plants (Fig. 2a). However only scattered and sparse colonies of PM had been established on leaves of the 35S-*VqWRKY31*-3 × Flag plants at this time.

Microscopic examination also revealed clear differences in PM disease progression between the 35S-*VqWRKY31-*3 × Flag and control plants. At 5 dpi, the epidermis of control leaves was covered with abundant hyphae and conidia (Fig. 2c). On the contrary, the germination of conidia on 35S-*VqWRKY31*-3 × Flag leaves was relatively restricted, and hyphae and conidia were rare. Moreover, staining with trypan blue, which is excluded from viable cells, revealed a punctate pattern on 35S-*VqWRKY31*-3 × Flag leaves, indicating isolated patches of cell death, while little or no cell death was apparent on control leaves. By 14 dpi, control leaves were almost completely covered with an abundant mass of spores, hyphae, and conidia, consistent with rapid progression of PM (Fig. 2c), while only sparse hyphae and conidia were found on the leaves of 35S-*VqWRKY31*-3 × Flag plants. The number of spores per milligram of inoculated leaves at 14 dpi was significantly greater in control plants as compared with 35S-*VqWRKY31*-3 × Flag plants (Fig. 2d). These results showed that *VqWRKY31* enhanced the resistance of *V. vinifera* to PM by restricting hyphal growth and sporulation.

To further evaluate the function of *VqWRKY31* in resistance to PM, we assessed PM disease progression after *E. necator* inoculation of ‘Shang-24’ plants in which *VqWRKY31* had been transiently silenced. In this experiment, the pART27-*VqWRKY31* RNAi vector was introduced into *Agrobacterium* and infiltrated into ‘Shang-24’ leaves. Leaves infiltrated with *Agrobacterium* alone were used as the control. Expression of the *NPT II* gene was verified in infiltrated leaves, and this was found to be associated with reduced expression of *VqWRKY31* (Supplementary Fig. S3a and b). Direct observation of PM symptoms in ‘Shang-24’ leaves was complicated by their tomentose nature (Supplementary Fig. S3c). However, microscopic examination revealed a striking difference in PM progression between leaves of *VqWRKY31* RNAi and control plants. In control leaves, most of conidia had not germinated by 3 dpi, whereas in *VqWRKY31* RNAi-silenced leaves hyphae had already appeared (Supplementary Fig. S3d). The hyphal length per colony for control leaves was substantially shorter than in *VqWRKY31* RNAi-silenced leaves (Supplementary Fig. S3e). These results showed that *VqWRKY31* positively regulates grapevine PM disease resistance.

### Constitutive expression of *VqWRKY31* increased levels of SA and ROS

**Figure 3:**
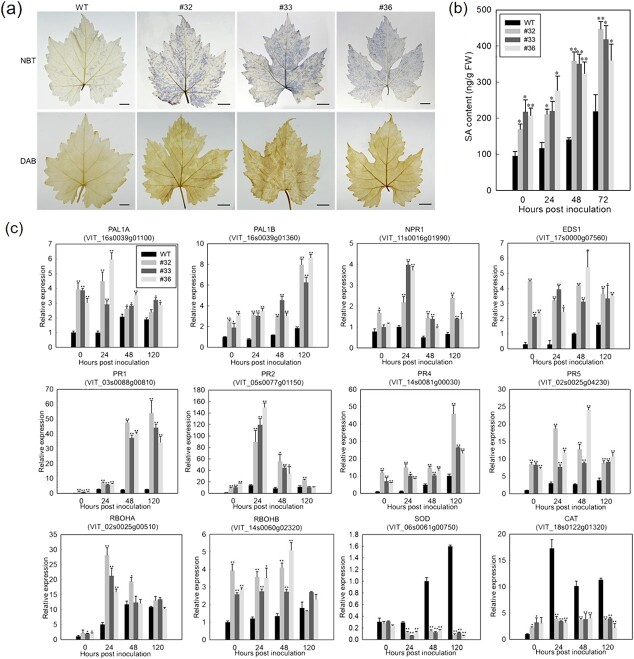
Expression of *VqWRKY31* increased the levels of SA and ROS. **a** NBT and DAB staining of leaves at 2 dpi (bar = 1 cm). **b** Endogenous SA content after inoculation. Data represent means ± standard errors (*n* = 3). Asterisks represent significant differences (^*^P < 0.05, ^**^P < 0.01, Student’s *t* test) between *VqWRKY31*-expressing lines and non-transgenic control. **c** Relative expression of genes related to SA signaling and ROS accumulation as analyzed by qRT–PCR in leaves after inoculation. The endogenous control used was *ACTIN7* (XM_002282480). Data represent means ± standard errors (*n* = 3). Asterisks represent significant differences (^*^P < 0.05, ^**^P < 0.01, Student’s *t* test).

SA is important for resistance against biotrophic pathogens in the plant defense system, and may promote cell death [11]. We determined the endogenous levels of SA at four time points during PM progression in transgenic 35S-*VqWRKY31*-3 × Flag ‘Thompson Seedless’ plants and non-transgenic controls. The SA content in 35S-*VqWRKY31*-3 × Flag plants was higher than in controls at all four time points after inoculation, and showed an overall increasing trend (Fig. 3b). To identify a causal mechanism, we evaluated the expression levels of specific genes that are known to participate in SA biosynthesis. Two phenylalanine ammonia lyase (*PAL*) genes showed increased expression in 35S-*VqWRKY31*-3 × Flag plants (Fig. 3c). We also monitored the expression of SA defense marker genes in 35S-*VqWRKY31*-3 × Flag and control plants (Fig. 3c). The expression of *NONEXPRESSOR OF PR GENES1* (*NPR1*) was higher in the 35S-*VqWRKY31*-3 × Flag plants than in the non-transgenic controls at all three time points after inoculation, and peaked at 24 hours post-inoculation (hpi) (Fig. 3c). The expression of *ENHANCED DISEASE SUSCEPTIBILITY 1* (*EDS1*) was higher in 35S-*VqWRKY31*-3 × Flag plants than in control plants both at the time of inoculation and at all time points after inoculation (Fig. 3c). The expression levels of pathogenesis-related 1 (*PR1*), *PR2*, *PR4*, and *PR5* were also higher in 35S-*VqWRKY31*-3 × Flag lines than in controls before and after inoculation (Fig. 3c). These results indicated that SA biosynthesis and SA-associated immunity signaling pathway were activated by *VqWRKY31*.

Cell-death-related defense is associated with conjugation of SA and a burst of ROS production [11]. Given that 35S-*VqWRKY31*-3 × Flag plants exhibited heightened levels of necrosis, we investigated whether the enhanced resistance of 35S-*VqWRKY31*-3 × Flag plants to PM was related to ROS levels. Staining with nitroblue tetrazolium (NBT) and diaminobenzidine (DAB) revealed a strong increase in superoxide ion (O_2_^−^) and hydrogen peroxide (H_2_O_2_) accumulation, respectively, in the leaves of 35S-*VqWRKY31*-3 × Flag plants compared with control plants at 2 dpi, suggesting that expression of *VqWRKY31* conferred increased ROS production (Fig. 3a). Plant responses to pathogens include localized ROS ‘bursts’, provided in part by NADPH oxidase/respiratory burst oxidase homolog (RBOH) proteins [36]. We found that the transcriptional levels of the NADPH oxidase genes *VvRBOHA* and *VvRBOHB* were higher in 35S-*VqWRKY31*-3 × Flag plants than in controls after inoculation (Fig. 3c). In addition, the abundance of transcripts corresponding to superoxide dismutase (SOD) and catalase (CAT), two typical antioxidant enzymes involved in the elimination of ROS in plant cells, was strikingly higher in controls than in 35S-*VqWRKY31*-3 × Flag plants (Fig. 3c). These results indicated that expression of *VqWRKY31* facilitated a burst of ROS production as well as enhancing the SA defense signaling pathway, which may have contributed to the immune response to biotrophic pathogens.

### Multiple defense-related and metabolism-related genes were upregulated in 35S-*VqWRKY31*-3 × Flag plants

**Figure 4:**
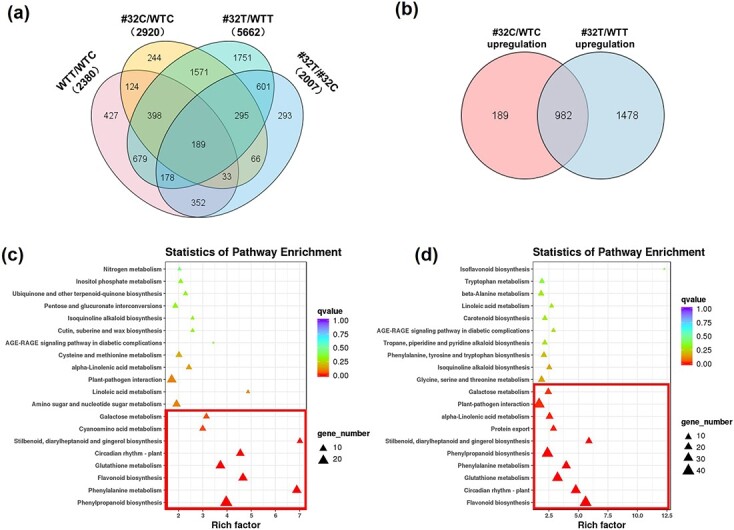
DEGs between *VqWRKY31*-expressing line #32 and non-transgenic controls. **a** Venn diagram showing the number of DEGs for *VqWRKY31*-expressing line #32 relative to controls at 0 and 24 hpi. WTC, WT plants at 0 hpi; WTT, WT plants at 24 hpi; #32C, #32 plants at 0 hpi; #32 T, #32 plants at 24 hpi. **b** Upregulated DEGs contained in #32C/WTC and #32 T/WTC. **c** Enriched KEGG pathways among upregulated genes of #32C/WTC. The significantly (*q* value < .05) changed pathways are enclosed by a red box. **d** Enriched KEGG pathways among upregulated genes of #32 T/WTT. The significantly (*q* value < .05) changed pathways are indicated by a red box.

Our results strongly implicated the *VqWRKY31* gene as an important factor in grapevine PM resistance. To investigate the molecular mechanism by which *VqWRKY31* may promote resistance, we used transcriptional profiling to compare gene expression profiles between 35S-*VqWRKY31*-3 × Flag (line #32) and control plants both before inoculation (0 hpi) and at 24 hpi. Differentially expressed genes (DEGs) were designated on the basis of at least a 2-fold difference in transcript abundance with false discovery rate (FDR) < .05. Using these criteria, a total of 2920 DEGs were identified at 0 hpi (#32C/WTC) and 5662 DEGs were identified at 24 hpi (#32 T/WTT) (Fig. 4a; Supplementary Tables S2 and S3). Among the 1171 genes that were found to be more highly expressed in 35S-*VqWRKY31*-3 × Flag plants than in controls at 0 hpi, 982 (83.9%) were also found to be more highly expressed in 35S-*VqWRKY31*-3 × Flag plants at 24 hpi, showing a significant overlap between the two sets of data (Fig. 4b).

To better understand the transcriptomic results, we analyzed the datasets with a standard KEGG (Kyoto Encyclopedia of Genes and Genomes) enrichment approach. This identified several significantly upregulated and downregulated pathways (*q* value < .05) between the 35S-*VqWRKY31*-3 × Flag and control plants (Fig. 4; Supplementary Fig. S4). Several pathways related to plant disease resistance or metabolism were upregulated in 35S-*VqWRKY31*-3 × Flag plants at 24 hpi; these included phenylpropanoid biosynthesis, flavonoid biosynthesis, plant–pathogen interaction and stilbenoid, diarylheptanoid, and gingerol biosynthesis (Fig. 4d). Moreover, many of the pathways found upregulated at 24 hpi were also upregulated prior to inoculation (Fig. 4c). These perturbations to disease-related and metabolic pathways further implicated VqWRKY31 as an important factor in PM disease resistance.

### Transgenic expression of *VqWRKY31* in grapevine altered the synthesis of specific metabolites

Transcriptomic analysis showed that the expression of multiple genes involved in metabolic pathways was altered in 35S-*VqWRKY31*-3 × Flag plants compared with controls under both *E. necator*-inoculated and non-inoculated conditions. To elucidate specific effects on metabolites, we carried out broadly targeted metabolome assays using an ultraperformance liquid chromatography-electrospray tandem mass spectrometry (UPLC-ESI-MS/MS)-based approach. Considering that these pathways were altered even in the absence of PM infection, we compared metabolite profiles and levels between 35S-*VqWRKY31*-3 × Flag and control plants in the absence of *E. necator* inoculation. Using this approach, we identified 168 metabolites whose levels were significantly higher in the 35S-*VqWRKY31*-3 × Flag plants (log_2_ fold change ≥1, VIP (variable importance in projection) ≥ 1), and 52 metabolites whose levels were significantly lower (Supplementary Table S4).

We then merged transcriptomic data and metabolomic data by mapping log-fold changes of transcripts and metabolites onto the KEGG pathways. Correlation analysis of the transcriptome and metabolome showed that only the flavonoid biosynthetic pathway and the stilbenoid, diarylheptanoid, and gingerol biosynthetic pathway were significant in KEGG enrichment analysis of differential genes and differential metabolites (*P* < .05) (Supplementary Figs S5 and S6). Thus, these two pathways were selected for further analysis.

**Figure 5:**
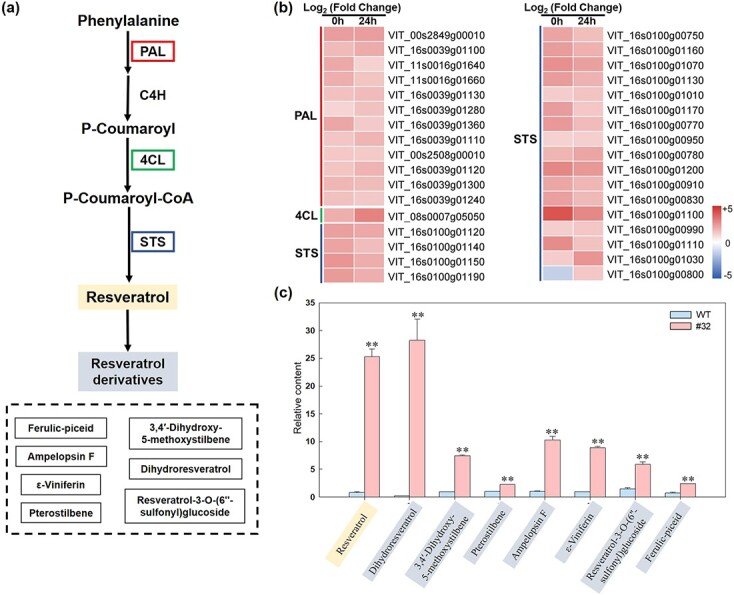
Correlation analysis of DEGs and metabolites involved in stilbene biosynthesis. **a** Simplified diagram of the stilbene biosynthetic pathway. PAL, phenylalanine ammonia-lyase; C4H, *trans*-cinnamate 4-monooxygenase; 4CL, 4-coumarate-CoA ligase; STS, stilbene synthase. **b** Heatmap analysis of the expression of DEGs involved in stilbene synthesis. Ratios of log_2_ fold changes are given as shades of red or blue colors. **c** Relative content of stilbene metabolites in the pathway. Data represent means ± standard errors (*n* = 3). Asterisks represent significant differences (^**^P < .01, Student’s *t* test) between *VqWRKY31*-expressing line #32 and control.

Phenylalanine ammonia-lyase (*PAL*), 4-coumaroyl-CoA ligase (*4CL*), and *STS* are key genes in the stilbenoid, diarylheptanoid, and gingerol biosynthesis pathways (Fig. 5a). We found that the expression of these genes in 35S-*VqWRKY31*-3 × Flag plants was increased after *E. necator* inoculation, and that expression of nearly all these genes (33 of 34) was also relatively higher in 35S-*VqWRKY31*-3 × Flag plants compared with controls in the absence of inoculation, implying that *VqWRKY31* is sufficient to activate these genes independently of other PM-associated signals (Fig. 5b). Consistent with the upregulation of key genes of the pathway, the content of stilbenes was relatively increased in 35S-*VqWRKY31*-3 × Flag plants compared with the non-transgenic controls. Of the 17 detected stilbene components, 8 showed increased content, while no stilbene content showed a decrease (Fig. 5c). The content of resveratrol, the core metabolite of the stilbenoid, diarylheptanoid, and gingerol biosynthesis pathway, was nearly 30-fold higher in 35S-*VqWRKY31*-3 × Flag plants compared with the non-transgenic controls (Fig. 5c), while the content of dihydroresveratrol was >700-fold higher (Fig. 5c). In addition, the content of resveratrol derivatives, such as ε-viniferin and pterostilbene, was significantly higher in the 35S-*VqWRKY31*-3 × Flag plants (Fig. 5c). These results strongly supported that *VqWRKY31* is a positive regulator of stilbenoids in grapevine.

**Figure 6:**
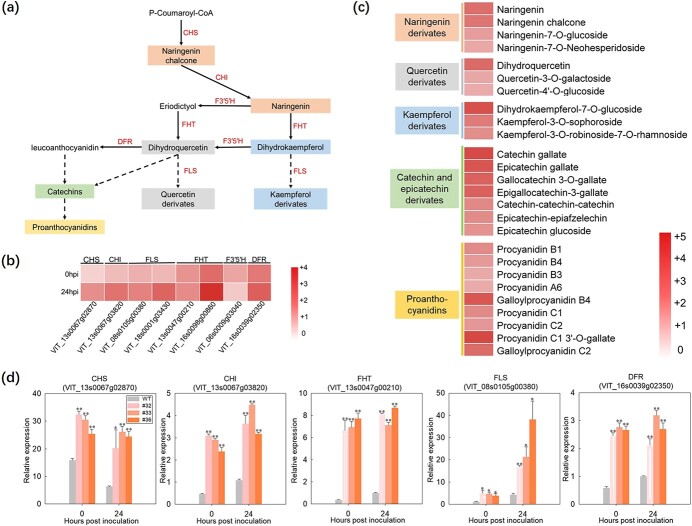
Correlation analysis of the DEGs and metabolites involved in flavonoid biosynthesis. **a** Simplified diagram of the flavonoid biosynthetic pathway. CHS chalcone synthase; CHI chalcone isomerase; F3′5′H flavonoid-3′,5′-hydroxylase; FLS flavonol synthase; FHT flavanone 3-dioxygenase; DFR dihydroflavanol 4-reductase. **b** Heatmap showing the log_2_ fold change of some key genes involved in flavonoid biosynthesis. **c** Heatmap showing the log_2_ fold change of related flavonoid derivatives between *VqWRKY31*-expressing and control plants. **d** Expression of genes participating in flavonoid biosynthesis after inoculation with *E. necator*. Expression was measured by qRT–PCR, using *ACTIN7* (XM_002282480) as an endogenous control. Data represent means ± standard errors (*n* = 3). Asterisks represent significant differences (^*^P < .05, ^**^P < .01, Student’s *t* test) between *VqWRKY31*-expressing and control lines.

We also noted that several metabolites associated with the flavonoid biosynthesis pathway, as well as the expression of several flavonoid biosynthetic genes, were higher in 35S-*VqWRKY31*-3 × Flag plants compared with the control. These included enzymes of flavonoid biosynthesis shared with the stilbenoid, diarylheptanoid, and gingerol biosynthesis pathway, such as PAL and 4CL (see above; Fig. 5b). However, expression of other key genes on the flavonoid pathway, including *CHS*, *CHI*, *F3′5′H*, *FHT*, and *FLS*, was higher in 35S-*VqWRKY31*–3 × Flag plants both under non-inoculated conditions and at 24 hpi (Fig. 6b and d). In addition, several important components of flavonoid synthesis, including naringenin chalcone, naringenin, quercetin derivates, kaempferol derivates, and catechin derivates, were also significantly increased in 35S-*VqWRKY31*-3 × Flag plants (Fig. 6c). A total of 39 flavonoids were increased significantly in 35S-*VqWRKY31*-3 × Flag plants compared with controls, whereas 17 flavonoids were decreased (Fig. 6c and; Supplementary Table S4). Proanthocyanidins, which are metabolites synthesized downstream of the flavonoid biosynthesis pathway, also showed general increases, with 9 of the 13 detected proanthocyanidins increased and none decreased (Fig. 6c; Supplementary Table S4). In summary, transgenic expression of *VqWRKY31* in ‘Thompson Seedless’ stimulated the synthesis of stilbenes, flavonoids, and proanthocyanidins.

### *VqWRKY31* directly regulated the expression of *STS* genes

**Figure 7:**
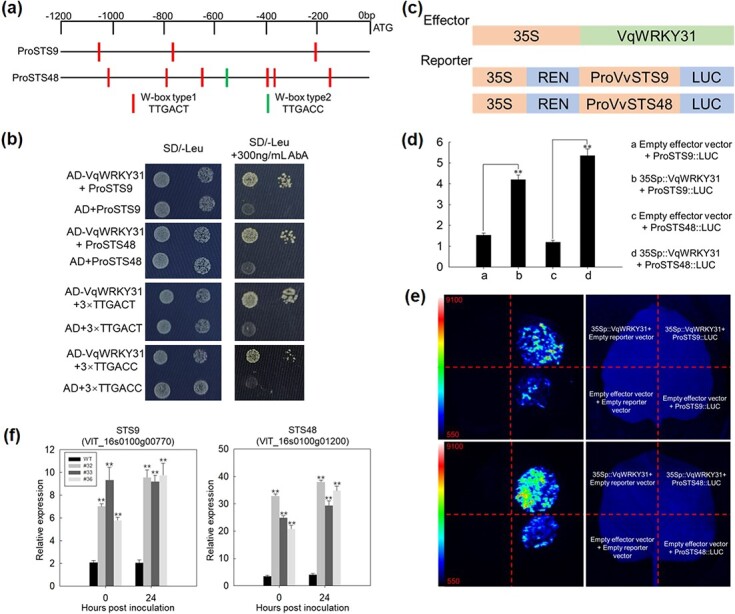
*VqWRKY31* upregulates the expression of *VvSTS9* and *VvSTS48*. **a** Schematic diagram of W-box type1 (TTGACT) and W-box type2 (TTGACC) in the promoters of *VvSTS9* and *VvSTS48*. **b** Yeast one-hybrid assay indicating that *VqWRKY31* can bind to the promoters of *VvSTS9* and *VvSTS48* and W-box. Yeast cultures were inoculated onto SD/−Leu media containing 300 ng/mL Aureobasidin A. **c** Schematic diagram of the effector and reporter constructs used for the dual-luciferase assay. **d**, **e** Luciferase (LUC) assay showing that *VqWRKY31* activates promoter activities of *VvSTS9* and *VvSTS48.* Data represent means ± standard errors (*n* = 3). Asterisks represent significant differences. **f** Relative expression of *STS9* and *STS48* was analyzed by qRT–PCR in leaves of transgenic and control plants after inoculation. *ACTIN7* (XM_002282480) was used as the reference gene. Data represent means ± standard errors (*n* = 3). Asterisks represent significant differences (^**^P < .01, Student’s *t* test) between *VqWRKY31*-expressing and control plants.

The transcriptomic and qRT–PCR results indicated that *VqWRKY31* can promote the expression of *STS* genes (Figs 5b and 7f). An analysis of *cis-*acting elements within the *V. vinifera STS* genes revealed that the promoters of *STS9* and *STS48* contained several W-box elements (TTGACT/C) (Fig. 7a; Supplementary Data S1). To support a role for *VqWRKY31* in activation of these two genes, we carried out a dual-luciferase reporter assay. The promoters of *STS9* and *STS48* were fused to a luciferase reporter and cotransformed with a 35S-*VqWRKY31* effector into tobacco leaf epidermal cells (Fig. 7c). Relative luciferase activities were determined, and the results showed that *VqWRKY31* can positively regulate the expression of *STS9* and *STS48 in vivo* (Fig. 7d and e).

We also took a yeast one-hybrid (Y1H) approach to evaluate the potential for VqWRKY31 to associate with the promoters of *STS9* and *STS48.* We transformed the pAbAi vectors containing promoter fragments from *STS9*, *STS48*, or a synthetic DNA containing three tandem repeats of the W-box type1 and W-box type2 elements into Y1HGold yeast as baits. The pGADT7 vector containing the CDS of *VqWRKY31* was used as prey. The results suggested that VqWRKY31 can directly associate with the promoters of *STS9* and *STS48*, and also that VqWRKY31 can bind to W-box type1 and W-box type2 elements (Fig. 7b).

## Discussion

PM is one of the most destructive diseases of grapevine, especially for *V. vinifera* cultivars that are most important for production of most wine and table grapes [1]. Given the adverse effects of fungicides on the environment, development of PM-resistant cultivars through molecular breeding is critical. However, there is relatively little knowledge about the genes conferring PM resistance and their associated transcriptional regulatory networks. Previous studies have shown that genes encoding transcription factors of the WRKY II group can be induced by pathogen inoculation and participate in immunity mechanisms in arabidopsis, *Glycine max*, *Malus domestica*, and *Solanum lycopersicum* [30, 37–39]*.* Here, we characterized a PM-induced gene encoding a WRKY IIb group transcription factor, *VqWRKY31*, from the PM-resistant species *V. quinquangularis*. When expressed in *V. vinifera*, *VqWRKY31* inhibited development of PM disease symptoms. Transgenic grapevine expressing *VqWRKY31* also showed increased SA immune signaling and ROS production. The combinatorial analysis of transcriptome and metabolome showed that *VqWRKY31* directed synthesis of multiple resistance-related metabolites, including stilbenes, flavonoids, and proanthocyanidins. These results indicate that *VqWRKY31* positively regulates grapevine resistance to PM via activating SA signaling and controlling disease-resistance-related metabolite synthesis.

To further investigate the potential role of *VqWRKY31* in resistance to PM, we generated transgenic *V. vinifera* constitutively expressing *VqWRKY31*. Stable transgenic lines were identified through PCR analysis of genomic DNA and western blot analysis. Western blotting identified a peptide with a molecular mass (~60.72 kDa) anticipated for the VqWRKY31-3 × Flag fusion protein (Fig. 1i). Interestingly, we noticed a second band that migrated more slowly in the gel (Fig. 1i). This may represent an unidentified, post-translationally modified form of VqWRKY31, as previously reported for WRKY transcription factors [40].

Several grapevine genes have been identified that are able to enhance PM resistance when expressed ectopically [1, 9, 16, 41]. In most cases, this is associated with reduced hyphal growth and spore density. Compared with non-transgenic control plants, *VqWRKY31*-expressing plants showed only limited development of PM and spore production, indicating a high resistance to PM (Fig. 2). Interestingly, leaves of *VqWRKY31*-expressing plants showed a punctate pattern with trypan blue staining, which may indicate localized cell death after *E. necator* inoculation (Fig. 2). Cell-death-related defense is important to limit the spread of PM and other biotrophic pathogens by initiating localized necrosis and blocking access of the pathogen to nutrients [11, 12]. Cell death is also a general distinguishing feature of highly PM-resistant grapevine genotypes [1, 35]. Moreover, many pathogen-related genes confer resistance against PM in grapevine through inducing cell death [1, 9, 16, 42]. Necrosis that occurred in the *VqWRKY31*-expressing lines was evident with a backlight, and a punctate pattern was conspicuous after trypan blue staining, suggesting that there may be a cell-death-related form of defense in transgenic grape (Fig. 2). However, we note that non-American grapevine species have not co-evolved with *E. necator*, and so the cell death observed in *E. necator*-inoculated *VqWRKY31*-expressing plants may be not a kind of HR related to effector-triggered immunity (ETI) [43].

SA signaling is a crucial component of the resistance response and programmed cell death [10]. Several studies have shown that, when inoculated with *E. necator*, PM-resistant grapevine genotypes accumulate endogenous SA to higher levels than PM-susceptible genotypes [35, 44]. Some proven PM-resistance genes can be induced by SA in grapevine [7, 9]. In addition, the activation of SA-dependent basal defense may be the basis of enhanced disease resistance to PM in Chinese wild grapevines [35]. Several WRKY transcription factors can mediate resistance to pathogens through the SA pathway [30, 37]. Our results also showed that the levels of endogenous SA in *VqWRKY31*-expressing plants were higher than that in non-transgenic controls (Fig. 3b), and that genes encoding the key enzyme of SA synthesis, PAL, were strongly induced in *VqWRKY31*-expressing lines (Fig. 3c). *NPR1* and *EDS1* are SA defense genes, which play important roles in plant defense [10, 45]. Several *PR* genes can also be induced by SA, including *PR1*, *PR2*, *PR4*, and *PR5* [46]. In this study, transcriptome and qRT–PCR assays showed that the *PR1*, *PR2*, *PR4*, *PR5*, and *EDS1* genes were induced in *VqWRKY31*-expressing plants from 0 to 120 hpi (Fig. 3c). *NPR1* was also upregulated in *VqWRKY31*-expressing plants after inoculation (Fig. 3c). Previous studies have documented that the role of SA in plant defense is often related to ROS, and that SA and ROS can synergistically drive the HR [11]. In this study, NBT and DAB staining revealed a burst of ROS production in the leaves of *VqWRKY31*-expressing lines (Fig. 3a). *VvRBOHA* and *VvRBOHB*, which are the main source of the oxidative burst in most plant–pathogen interactions [13], were also more highly expressed in *VqWRKY31*-expressing plants, whereas the enzymatic activities of SOD and CAT, two vital plant antioxidant enzymes, were significantly decreased (Fig. 3c) [13]. These results suggest that transgenic expression of *VqWRKY31* activated the SA defense signaling pathway and promoted ROS burst, contributing to PM resistance.

To further elucidate the molecular mechanism by which *VqWRKY31* enhanced resistance to PM, we conducted a transcriptome analysis of transgenic *VqWRKY31*-expressing and non-transgenic control plants. This revealed that multiple genes involved in plant defense and secondary metabolism were highly induced in the *VqWRKY31*-expressing plants. KEGG enrichment identified several upregulated pathways both at 24 and at 0 hpi, prior to pathogen treatment (Fig. 4c and d). We also compared genes upregulated both in inoculated *VqWRKY31*-expressing plants relative to inoculated controls and in non-inoculated *VqWRKY31*-expressing plants relative to non-inoculated controls. We identified 982 upregulated genes in both groups, indicating that their response to ectopic *VqWRKY31* was not dependent on the pathogen (Fig. 4b). This gene set contained *EDS1*, *PR2*, *PR4*, *PR5*, *STS*, *PAL*, *CHI*, *FLS*, *FHT*, and additional genes documented as important to plant defense and secondary metabolism. SA content in *VqWRKY31*-expressing lines was also increased relative to controls at 0 hpi (Fig. 3b). Various studies have shown that some highly PM-resistant species of Chinese and North American wild grapevines have ‘resistance reserves’. For example, SA content in Chinese *Vitis pseudoreticulata* ‘Baihe-35-1’ and North American *Vitis aestivalis* was higher than that in *V. vinifera* even in the absence of pathogens [35]. In ‘Baihe-35-1’ and another wild Chinese accession, ‘Baishui-40’, pathogen resistance-related genes such as *EDS1*, *PR1*, *PAL*, and *PR3* also exhibited elevated transcript level in comparison with ‘Thompson Seedless’ under natural conditions [35]. In *V. aestivalis*, *EDS1*, *PR1*, *PR2*, *PR3*, *PR9*, and *STS*, as well as specific genes participating in secondary metabolism, were expressed at significantly higher levels compared with *V. vinifera* ‘Cabernet Sauvignon’ [44]. The constitutively elevated expression of defense and metabolism genes may be an important strategy against PM in resistant grapevine species.

Moreover, the transcriptomic results also showed that a large number of genes in secondary metabolism pathways were expressed to higher levels in *VqWRKY31*-expressing plants relative to controls both before and after pathogen inoculation, which may be crucial for enhanced PM resistance. We conducted a metabolomic assay and merged it with the transcriptomic data, and two important pathways were highlighted: the flavonoid biosynthetic pathway and the stilbenoid, diarylheptanoid, and gingerol biosynthesis pathway (Supplementary Fig. S6). Stilbenes are key metabolites related to the stilbenoid, diarylheptanoid, and gingerol biosynthesis pathway. As phytoalexins, stilbenes play important roles in plant protection against pathogens [14]. The content of stilbenes can be induced by infection with pathogens, including *E. necator* [18]. The precursor of other stilbenes, resveratrol, increased by ~30-fold in the strongly *VqWRKY31*-expressing line #32 (Fig. 5c). Resveratrol is the precursor of many derivatives, such as ε-viniferin, pterostilbene, and piceid [47]. Many studies recognized that resveratrol, ε-viniferin, and pterostilbene can enhance the resistance of grapevine to fungal disease by inhibiting the production and germination of spores and restraining pathogen development [18, 48]. Tissue necrosis after pathogen inoculation correlated with stilbene phytoalexin accumulation, and stilbene phytoalexin could also initiate cell death in grapevine [18, 49]. Unlike ε-viniferin and pterostilbene, which are fungal toxins, piceid is considered a non-toxic compound [50]. Previous studies showed that piceid accumulated primarily in susceptible grapevine cultivars, whereas fungitoxic stilbenes mainly accumulated in resistant cultivars [18]. Similar results were also observed in our study: the accumulation of resveratrol, ε-viniferin, and pterostilbene was significantly promoted in *VqWRKY31*-expressing plants, whereas piceid was not significantly changed (Fig. 5c; Supplementary Table S4). We observed limited growth of *E. necator* in *VqWRKY31*-expressing plants, which is consistent with the fungitoxicity of stilbenes, underscoring the relationship between induced toxic stilbene accumulation and enhanced pathogenic resistance in the transgenic plants.

Due to the accumulation of high levels of stilbenes in *VqWRKY31-*expressing plants, we tested whether *VqWRKY31* can regulate the expression of key enzymes in stilbene synthesis. STS is a core enzyme family catalyzing the formation of the stilbene backbone [18]. In the *V. vinifera* genome, 48 *STS* genes were identified [51], and previous research demonstrated that they play a positive role in plant defense. The ectopic expression of grapevine STS in arabidopsis and barley enhanced their resistance against fungal disease [15, 16], whereas overexpression of an *STS* gene in grapevine promoted accumulation of stilbenes, including resveratrol, and tolerance to PM [16]. Some transcription factors are known that can directly or indirectly regulate the expression of STS. *VvMYB14* and *VvMYB15* are positive regulators of *STS* gene(s), and the ectopic expression of *MYB15* in hairy roots can increase stilbene accumulation [52]. *VdMYB1* promoted the expression of STS, and transient expression of *VdMYB1* enhanced resistance to fungal pathogens in grapevine [7]. VqERF114 can interact with VqMYB35 to activate the expression of STS and stilbene synthesis [53]. In addition, the grapevine WRKY transcription factor, VqWRKY53, of the WRKY IIc group, can directly induce the expression of STS and enhance disease resistance to *Pst* DC3000 when expressed in arabidopsis [8]. A previous study also suggested that WRKY transcription factors may regulate the expression of STS in grapevine [43]. In this study, corresponding to the observed increase of resveratrol and stilbenes, the relative expression of most *STS* genes was activated in *VqWRKY31*-expressing plants (Fig. 5b). Our study showed that *VqWRKY31* can directly bind to the two W-box (TTGACT/C) elements and the promoters of *STS9* and *STS48*, thereby activating the expression of these two *STS* genes (Fig. 7). Other key enzymes in stilbene synthesis, such as PAL and 4CL, were also highly induced in *VqWRKY31*-expressing plants (Fig. 5b). Therefore, *VqWRKY31* directly induced STS expression and activated genes among multiple processes of stilbene synthesis, showing its comprehensive regulation of stilbene synthesis.

Multiomics data also suggested that expression of *VqWRKY31* also altered flavonoid biosynthesis. Genes encoding catalytic enzymes in flavonoid biosynthesis, such as CHS, CHI, FHT, F3′5′H, and FLS (Fig. 6a) [25, 26], were more strongly expressed in *VqWRKY31*-expressing plants relative to controls, which was consistent with the significant difference in flavonoid content in the *VqWRKY31*-expressing plants (Fig. 6). CHS and CHI are the first two enzymes in the flavonoid biosynthetic pathway, and catalyze the production of naringenin chalcone and naringenin, which have antimicrobial effects on fungal and bacteria [54, 55]. The content of naringenin chalcone and naringenin was increased in *VqWRKY31*-expressing plants (Fig. 6c). Quercetin derivatives, including dihydroquercetin, quercetin-3-*O*-galactoside, and quercetin-4′-*O*-glucoside, also accumulated to higher levels in *VqWRKY31*-expressing plants compared with controls (Fig. 6c). In some previous studies, quercetin derivatives have shown toxic effects against pathogens. Quercetin-3-galactoside has antifungal activity *in vitro* [56], and dihydroquercetin participates in the defense system against *Fusarium* species and *Xanthomonas oryzae* [55]. Quercetin can also serve as a prooxidant. It can induce resistance to *Pst* DC3000 in arabidopsis by causing an H_2_O_2_ burst and cell death [20]. SA signaling and expression of *PR1* and *PAL1* genes can also be enhanced after quercetin treatment in arabidopsis plants challenged with virulent *P. syringae* pv. Tomato [20]. Kaempferol derivatives, including kaempferol-3-*O*-robinoside-7-*O*-rhamnoside, kaempferol-3-*O*-sophoroside, and dihydrokaempferol-7-*O*-glucoside, were also highly enriched in the *VqWRKY31*-expressing plants (Fig. 6c). These metabolites often serve as a positive regulator of plant defense [22, 57]. In addition, catechins, a class of secondary metabolites with antimicrobial activity [21, 22], were also present at increased levels in *VqWRKY31*-expressing plants (Fig. 6c). Previous studies have shown that catechin derivatives inhibit fungal diseases in satsuma orange, Norway spruce, and arabidopsis [58–60].

Additionally, proanthocyanidins, which are oligomers or polymers composed of flavonoid units, were also relatively highly abundant in the *VqWRKY31*-expressing line #32 (Fig. 6c). As a kind of tannin, proanthocyanidins play an important role in plant defense against fungal diseases [61]. In strawberry, accumulation of proanthocyanidins can be induced by SA and can enhance resistance to *Podosphaera aphanis* [23]. In poplar, *MYB115* regulates proanthocyanidin biosynthesis to enhance fungal resistance [62]. In *Populus trichocarpa*, proanthocyanidin accumulation and fungal resistance can also be enhanced by constitutive expression of *PtrLAR3* [61]. Previous studies found that several transcription factors in grapevine and other plants regulate proanthocyanidin synthesis, but most of these belong to the MYB or bHLH class [63–65]. In this study, we found that *VqWRKY31*, as a WRKY transcription factor, plays a positive regulatory role in the synthesis of proanthocyanidins in grapevine.

**Figure 8:**
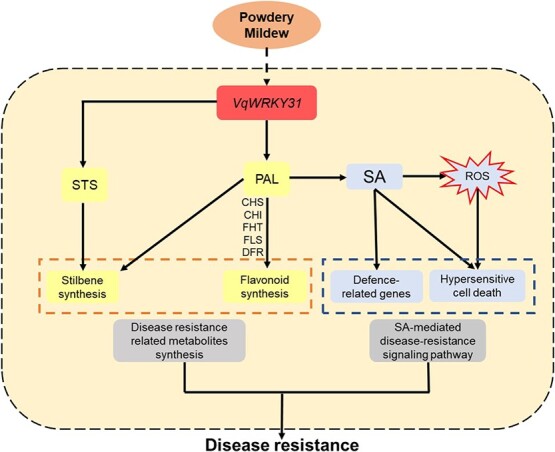
Model for the role of *VqWRKY31* in resistance to PM in grapevine. *VqWRKY31* facilitates the transcript abundance of specific genes, enhancing SA accumulation, ROS production, and stilbene and flavonoid synthetic pathways, thereby increasing resistance-related metabolite synthesis, activating SA defense signaling, and finally enhancing PM resistance. Arrows represent the facilitated effect.

In conclusion, we determined the mechanism of *VqWRKY31* in promoting PM resistance. Our results indicate that *VqWRKY31* enhances SA accumulation and ROS, resulting in increased expression of pathogen-related genes. *VqWRKY31* also promotes the content of resistance-related stilbene and flavonoid derivatives. These two pathways of disease-resistance-related metabolite synthesis and SA defense signaling both contributed to the strong resistance of *VqWRKY31*-expressing plants to PM (Fig. 8).

## Materials and methods

### Plant materials

*V. vinifera* L. cv. ‘Thompson Seedless’ and *V. quinquangularis* accession ‘Shang-24’ were maintained in the grapevine germplasm resource of Northwest A&F University, Yangling, Shaanxi, China. Transgenic and non-transgenic control grapevine seedlings obtained by plant tissue culture were grown in a controlled-environment chamber under 16-hours light/8-hours dark photoperiods at 25°C for 2 months. Then, they were moved to a greenhouse and grown at 21–26°C for 2 months before use in experiments. Tobacco (*Nicotiana benthamiana*) plants were grown in a growth chamber at 25°C under 16-hours light/8-hours dark photoperiods.

### Bioinformatic analyses

The phylogenetic analysis of VqWRKY31 and other WRKY proteins was done using MEGA 5.0. The amino acid sequence alignment between VqWRKY31 and related WRKY proteins was conducted using DNAMAN.

### Analysis of subcellular localization

The CDS of *VqWRKY31* without the stop codon was cloned by PCR using the oligonucleotide primers GFP-*WRKY31* forward and GFP-*WRKY31* reverse (Supplementary Table S1) and then inserted into the pCAMBIA2300-GFP vector. The recombinant vector 35S-*VqWRKY31*-GFP and the non-modified pCAMBIA2300-GFP vector were introduced into *Agrobacterium tumefaciens* strain GV3101 and transformed into tobacco leaf epidermal cells as previously reported [30]. DAPI (4′,6-diamidino-2-phenylindole) solution (Solarbio, Beijing) was infiltrated into tobacco leaves for 5–10 minutes before observation. The fluorescence signal was detected using a laser confocal microscope (TCS-SP8 SR, Leica, Germany) under 40× magnification. The excitation wavelengths used were 405 nm for DAPI and 488 nm for GFP, and the emission wavelengths were collected between 415 and 435 nm for DAPI and between 500 and 530 nm for GFP.

### Generation of *VqWRKY31*-overexpressing transgenic plants and transient transformation

The *VqWRKY31* cDNA was cloned by PCR using the primers 2300flg*-WRKY31* (forward) and 2300flg*-WRKY31* (reverse) (Supplementary Table S1) and then inserted into the pCambia2300 vector downstream of the CaMV35S promoter to generate CaMV35S-*VqWRKY31*-3 × Flag. *A. tumefaciens* GV3101 harboring 35S-*VqWRKY31*-3 × Flag was used for the stable transformation of grapevine via previously described methods [31]. Young vegetative shoots from transgenic plants were transferred to agar-solidified MS medium every 2 months for rapid propagation.

Sense and antisense fragments from *VqWRKY31* (~300 bp) were amplified by PCR using the primers FWRKY31-RNAi-sense, RWRKY31-RNAi-sense, FWRKY31-RNAi-antisense, and RWRKY31-RNAi-antisense (Supplementary Table S1) and then inserted into the pKANNIBAL vector. The modified pKANNIBAL plasmids were digested with NotI to generate linear expression cassette fragments. These cassettes were inserted into the pART27 vector to generate the *VqWRKY31* RNAi vector. This was introduced into *V. quinquangularis* ‘Shang-24’ via *Agrobacterium*-mediated transient transformation.

For transient expression assays, cultures of *A. tumefaciens* GV3101 carrying the appropriate plasmid vectors were incubated at 28°C with shaking at 200 rpm for 12 hours, and cells were collected by centrifugation for 10 minutes at 12 000 rpm. Cells were resuspended in 10 mM MES containing 10 mM MgCl_2_ and 200 μM acetosyringone, pH adjusted to 5.6, and cells were adjusted to an OD_600_ of 0.6. Detached leaves were gently rinsed in sterile water, and then submerged in bacterial suspension. Afterwards, leaves were subjected to a vacuum at 0.085 MPa for 30 minutes. After vacuuming, excess bacterial suspension was removed by blotting, and leaves were placed in a tray with the petioles wrapped in moist cotton. The tray was sealed with preservative film to maintain the moisture level. Expression of the *NEOMYCIN PHOSPHOTRANSFERASE II* (*NPT II*) gene was detected using qRT–PCR and primers as listed in Supplementary Table S1.

### Identification of transgenic plants

Genomic DNA was extracted from grapevine leaves using a commercial kit (DP320, Tiangen Biotech, China). The presence of the T-DNA insertion in *VqWRKY31*-overexpressing lines was evaluated using PCR and the primers 2300-flg (forward) and 2300-flg (reverse). DNA from non-transgenic, wild-type control plants served as a negative control.

Western blot analysis of transgenic lines was carried out to confirm the expression of VqWRKY31-3 × Flag protein. Fresh leaves were ground into powder using a mortar and pestle under liquid nitrogen, and ~ 300 mg powdered tissue was homogenized in extraction buffer as described [32]. Then, the samples were subjected to centrifugation at 12 000 × g for 10 minutes. The supernatant was mixed with SDS–PAGE sample loading buffer and placed in a boiling water bath for 5 minutes. Western blotting was conducted as previously described [32]. Anti-Flag and peroxidase-conjugated goat anti-mouse IgG (H + L) were used for immunoblotting.

### Quantitative real-time PCR

Total RNA was isolated from young leaves using a commercial kit (Plant RNA Kit; R6827-01; Omega Bio-Tek, USA). Reverse transcription was carried out using Prime Script TMR Tase (TaKaRa Biotechnology, Dalian, China). qRT–PCR was performed on a StepOnePlus Real-Time PCR System (Applied Biosystems, USA) with SYBR Green according to the user manual (TaKaRa Biotechnology, Dalian, China). The endogenous control used was *ACTIN7* (XM_002282480). Specific primers used in this assay are listed in Supplementary Table S1. All experiments were carried out with three independent replicates.

### Pathogen infection, histochemical staining, and microscopic analysis

*E. necator* cultures were maintained on *V. vinifera* L. cv. ‘Thompson Seedless’ in a greenhouse at 26°C to generate fresh conidia. One-year-old leaves were inoculated with fresh conidia as previously described [33]. At 5 and 14 days after inoculation, leaves were submerged in lactophenol–trypan blue solution (20 ml ethanol, 10 ml phenol, 10 ml lactic acid, and 10 mg trypan blue dissolved in 10 ml sterile water) in glass containers. Afterwards, containers were subjected to a vacuum at 0.085 MPa for 30 minutes and then heated to boiling for 5 minutes. After cooling to room temperature, leaves were decolorized in 2.5 g ml^−1^ chloral hydrate solution for 24 hours. PM incidence was observed with an automated fluorescence microscope (BX63, Olympus, Japan) under bright-field 4× and 10× magnification. Infected leaves were cut and weighed, and 2 mg of leaf pieces was soaked in 2 ml sterile water with gentle agitation. Spores were counted with an automated fluorescence microscope (BX63, Olympus, Japan). DAB and NBT staining were performed as previously described [34].

### Salicylic acid measurements

Leaves from transgenic or control plants were collected at four time points after *E. necator* inoculation and placed in liquid nitrogen. Extraction of SA was performed as previously described [35]. SA content was measured using a triple-quadrupole LC/MS system (1290 Infinity II-6470, Agilent Technologies, USA) as previously described [35]. All experiments were carried out with three independent replicates.

### Transcriptomic analysis

Leaves from the *VqWRKY31*-overexpressing line #32 or control plants were collected at 0 and 24 hours after *E. necator* inoculation. Total RNA was isolated from young leaves using a commercial kit (Plant RNA Kit; R6827-01; Omega Bio-Tek, USA). Sequencing libraries were constructed using the NEBNext Ultra™ RNA Library Prep Kit for Illumina (NEB, USA). RNA libraries were sequenced using the Illumina HiSeq 2500 platform by BioMarker Technologies (Beijing). Reads per kilobase per million mapped reads (RPKM) values were calculated to normalized expression data, and the DESeq2 R package was used to analyze differential expression among samples. DEGs were defined as undergoing absolute fold change ≥2 with FDR <.05. KOBAS [66] software was employed for KEGG enrichment analysis of DEGs.

### Metabolite extraction, measurement, and analysis

Leaves from 1-year-old *VqWRKY31*-overexpressing or control plants were collected in the same growth state as the plants used for pathogen inoculation and transcriptomic analysis, and then freeze-dried using a vacuum freeze-dryer (Scientz-100F, Scientz, China). Then, the samples were subjected to metabolome analysis [67] using an UPLC-ESI-MS/MS system from Metware Biotechnology. Metabolites with VIP (variable importance in the projection) ≥1 and absolute log_2_ fold change ≥1 were considered differentially regulated.

### Yeast one-hybrid assay

The promoter regions of *VvSTS9* (1185 bp) and *VvSTS48* (1205 bp) were amplified by PCR and inserted into the pAbAi vector to generate pAbAi-*VvSTS9* and pAbAi-*VvSTS48*, respectively. Three tandem copies of TTGACC (W-box type 1) and TTGACT (W-box type 2) were also inserted into the pAbAi vector. These constructions were digested by the restriction enzyme BbsI (NEB, USA) for linearization and then transformed into the Y1HGold yeast strain for use as bait. The CDS of *VqWRKY31* was cloned into pGADT7 to generate AD-*VqWRKY31* as prey, and this was transformed into bait strains. Single colonies were selected and grown on SD/−Leu media with 300 ng/mL Aureobasidin A to confirm positive interactions. Bait strains transformed with the non-modified pGADT7 vector served as the negative control. Specific primers used in these constructions are listed in Supplementary Table S1.

### Dual-luciferase reporter assay

The promoter sequences containing the W-box (TTGACC or TTGACT) of *VvSTS9* and *VvSTS48* were inserted into the pGreen II 0800-LUC vector to serve as reporters, and the CDS of *VqWRKY31* was inserted into the pGreen II 62-SK vector to serve as the effector. Specific primers used in these constructions are listed in Supplementary Table S1. These plasmids were transformed into *A. tumefaciens* GV3101 along with the helper plasmid pSoup-p19 (#AC1003, Weidi, Shanghai, China). Four-week-old tobacco leaves were co-infiltrated with *Agrobacterium* harboring the effector plasmid and different reporter plasmids as described previously [32]. Dual-luciferase assay reagents (Promega) were used to measure the activities of firefly luciferase and *Renilla* luciferase on an Infinite M200 PRO enzyme labeling instrument (Tecan, Hombrechtikon, Switzerland) as described [39]. All experiments were conducted with three independent replicates.

## Supplementary Material

Web_Material_uhab064

## Data Availability

The raw sequencing data have been deposited in NCBI SRA under the accession number SRP337143.
