# Supplementary figures and images for: Overexpression of VqWRKY31 enhances powdery mildew resistance in grapevine by promoting salicylic acid signaling and specific metabolite synthesis
Source: Hortic Res. 2022 Jan 19;9:uhab064. doi: 10.1093/hr/uhab064 (PMC8944305; doi:10.1093/hr/uhab064)

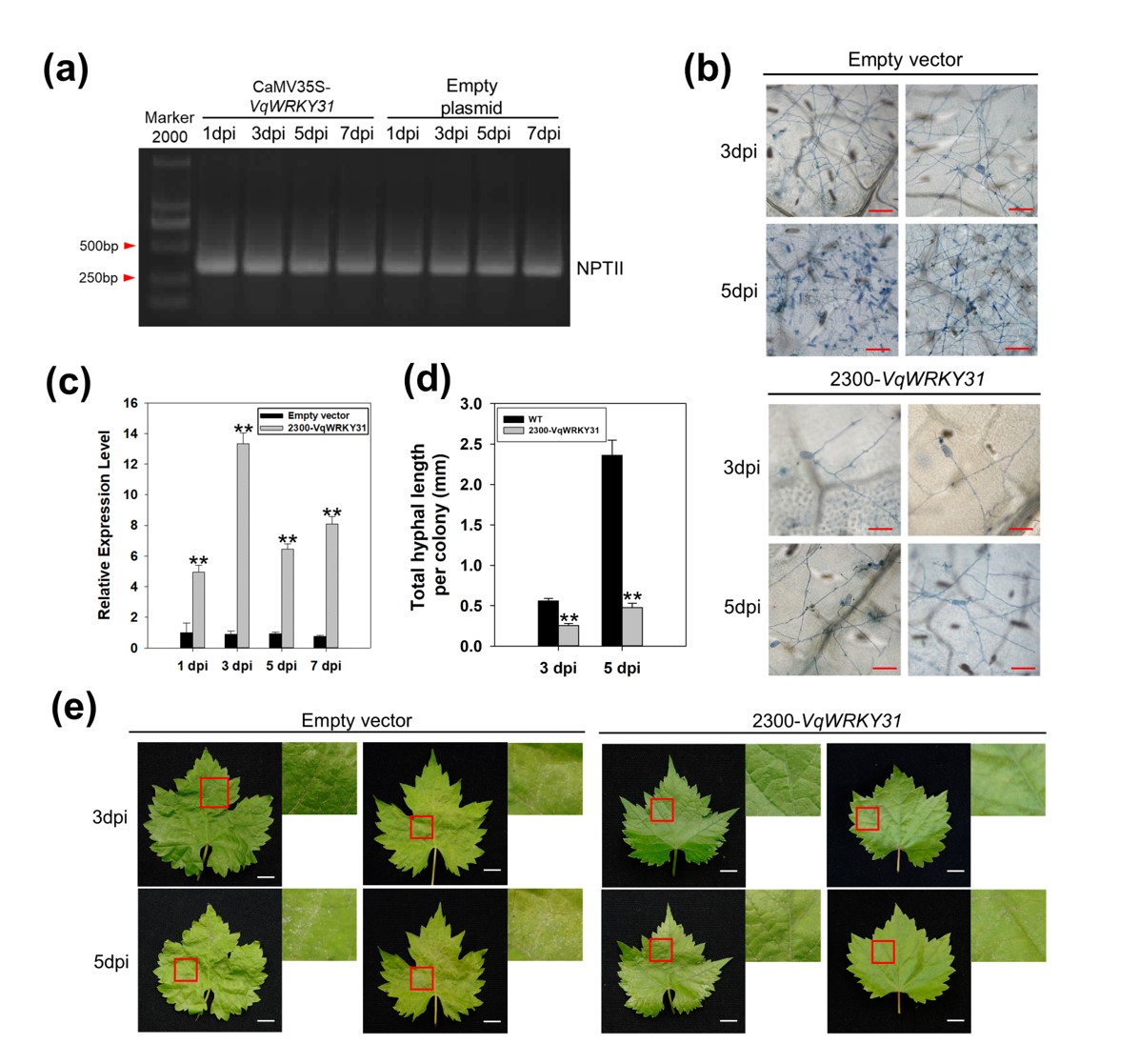

Supplement: Web_Material_uhab064 [file web_material_uhab064.zip › Fig. S1.jpg]

**(a)**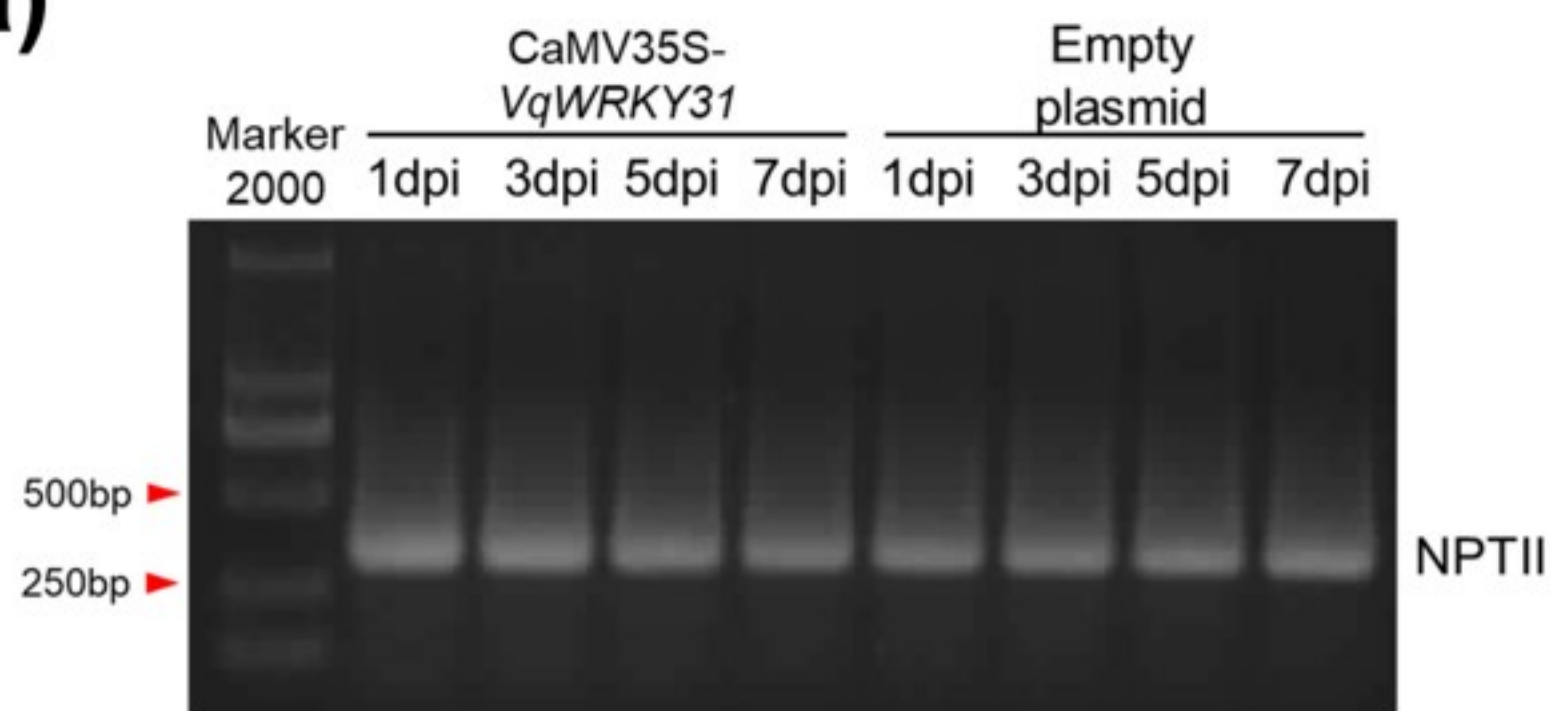**(b)**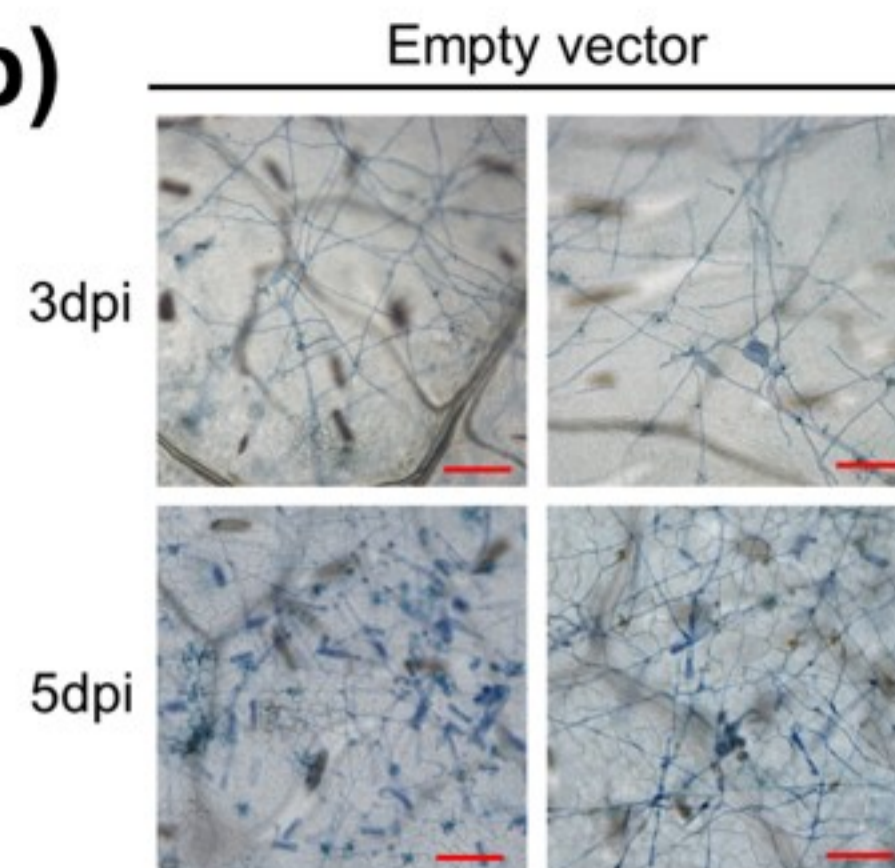**(c)**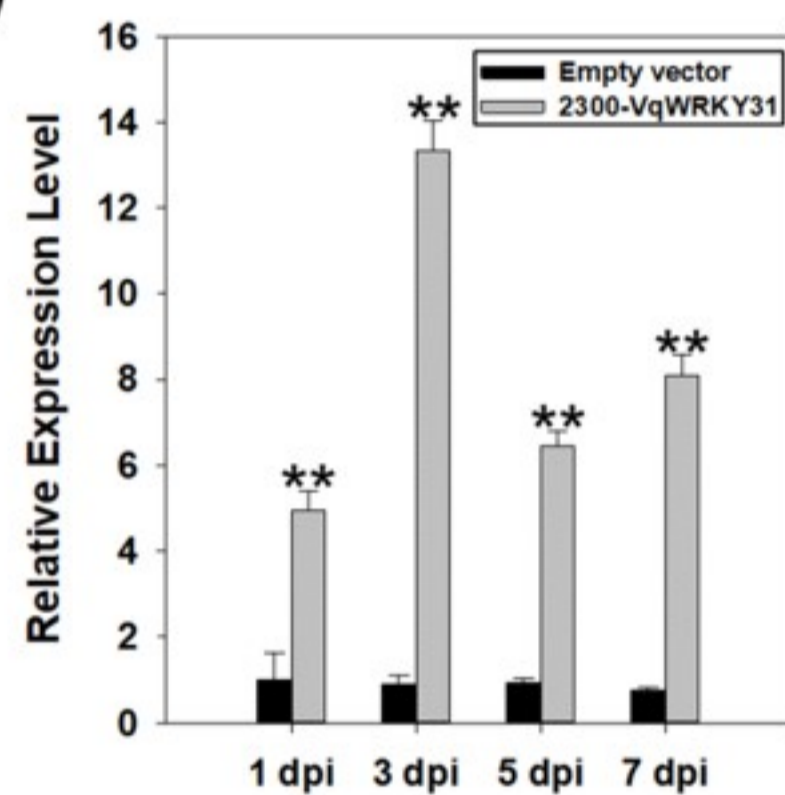**(d)**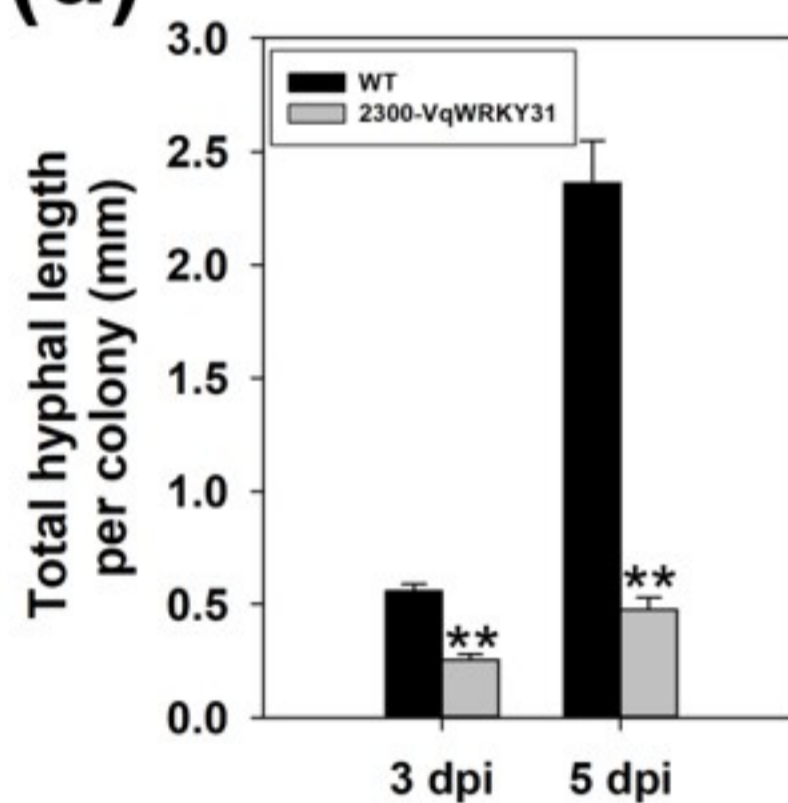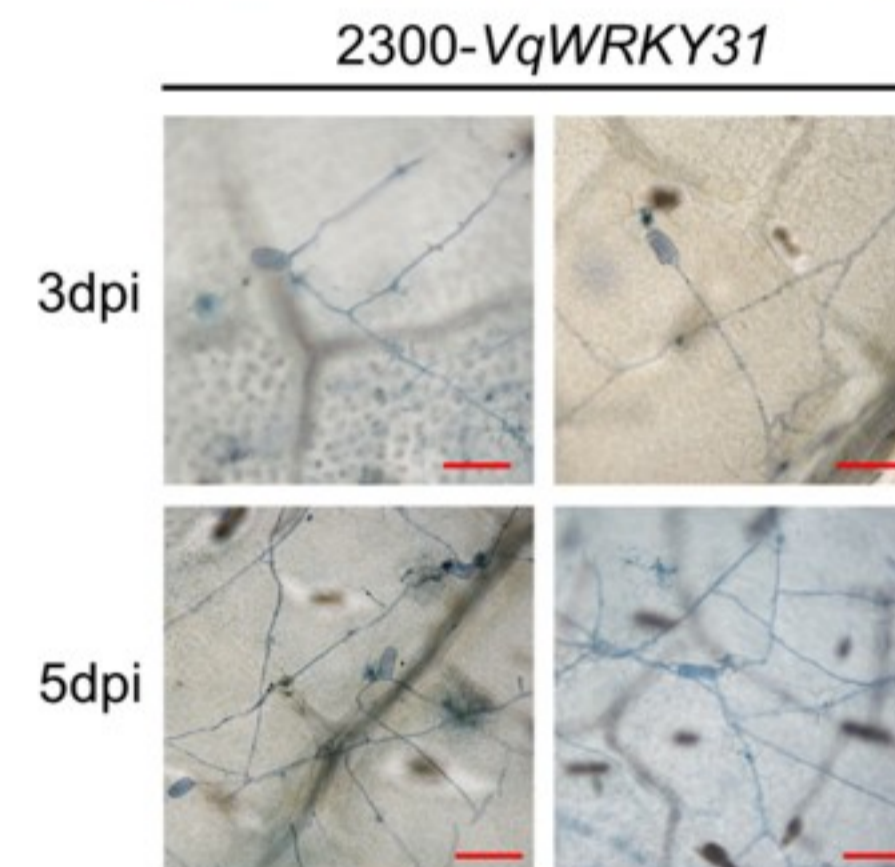**(e)**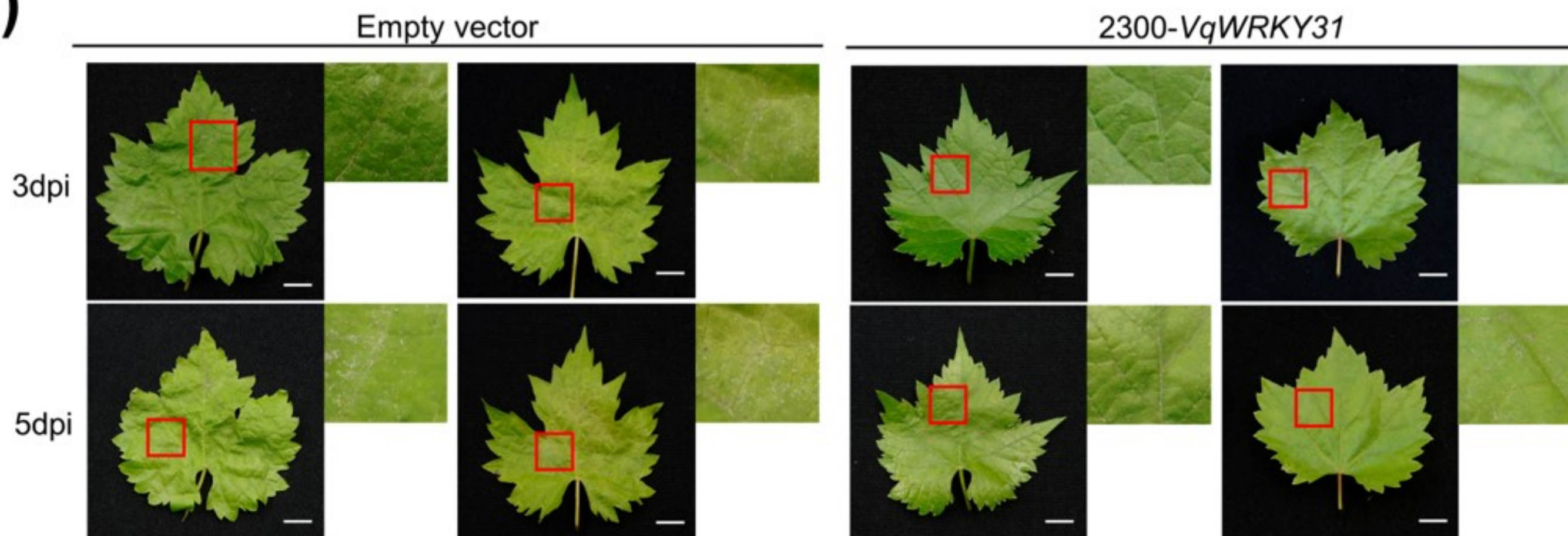

Supplement: Web_Material_uhab064 [file web_material_uhab064.zip › Fig. S1.pdf]

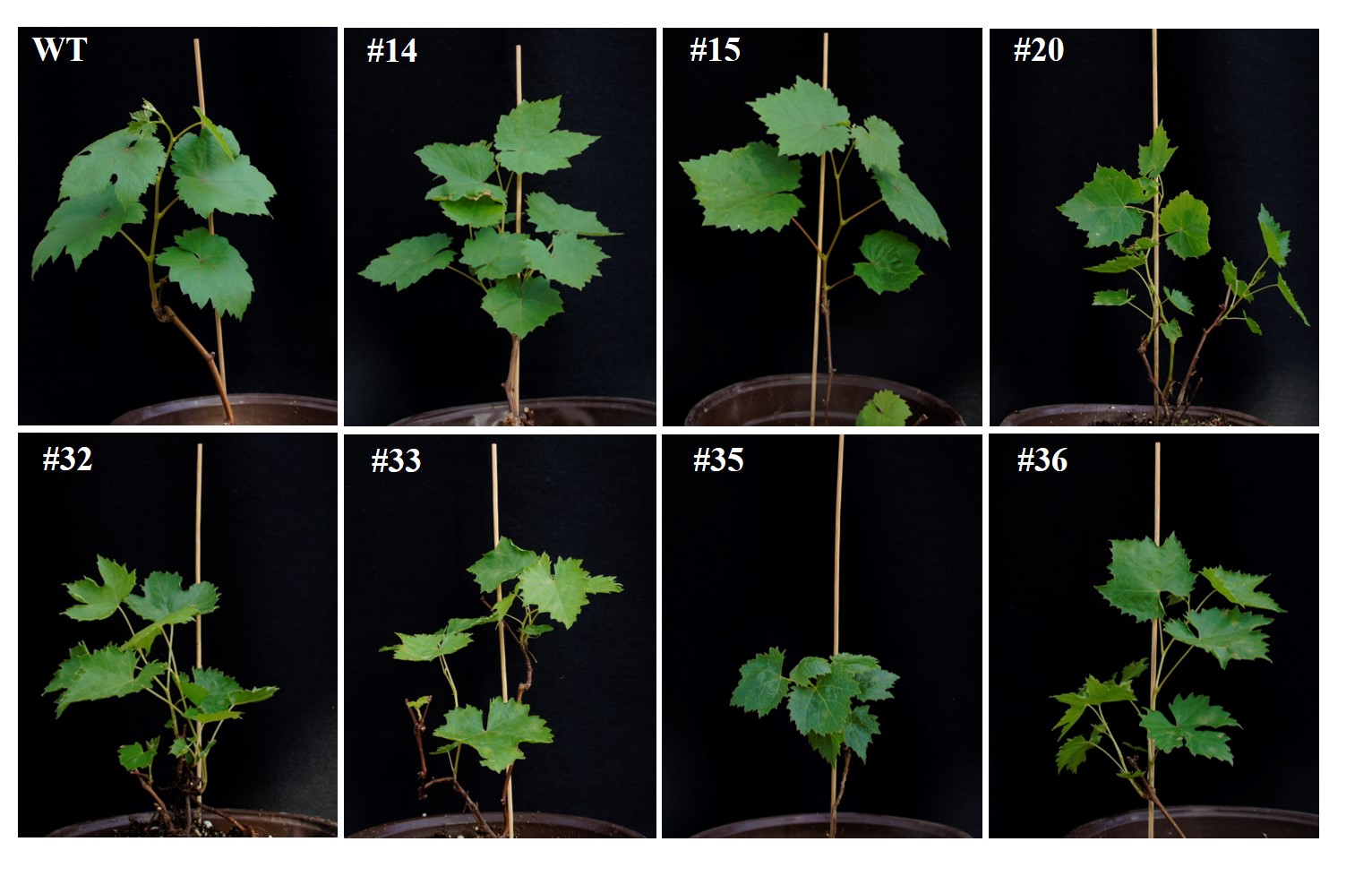

Supplement: Web_Material_uhab064 [file web_material_uhab064.zip › Fig. S2.jpg]

**WT**

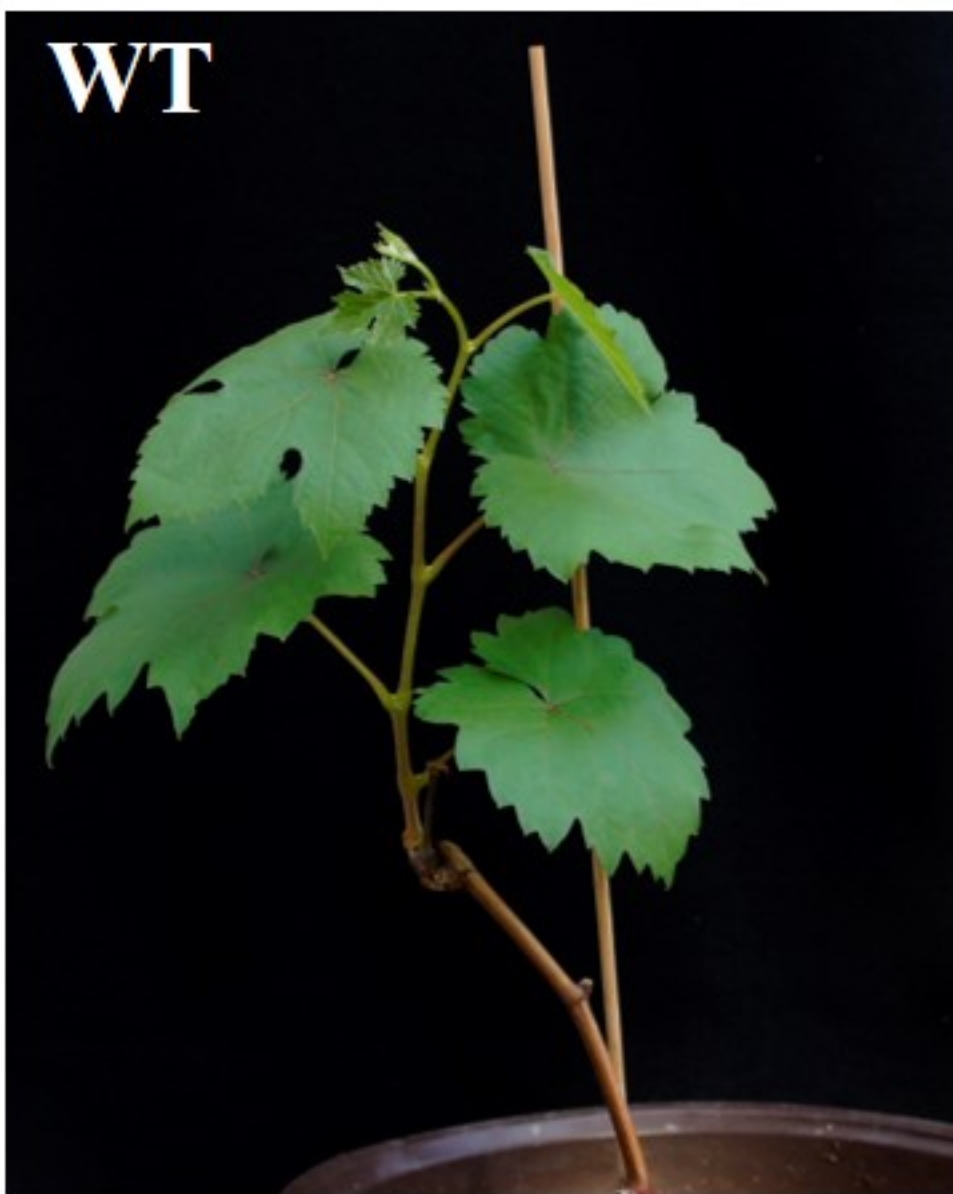

**#14**

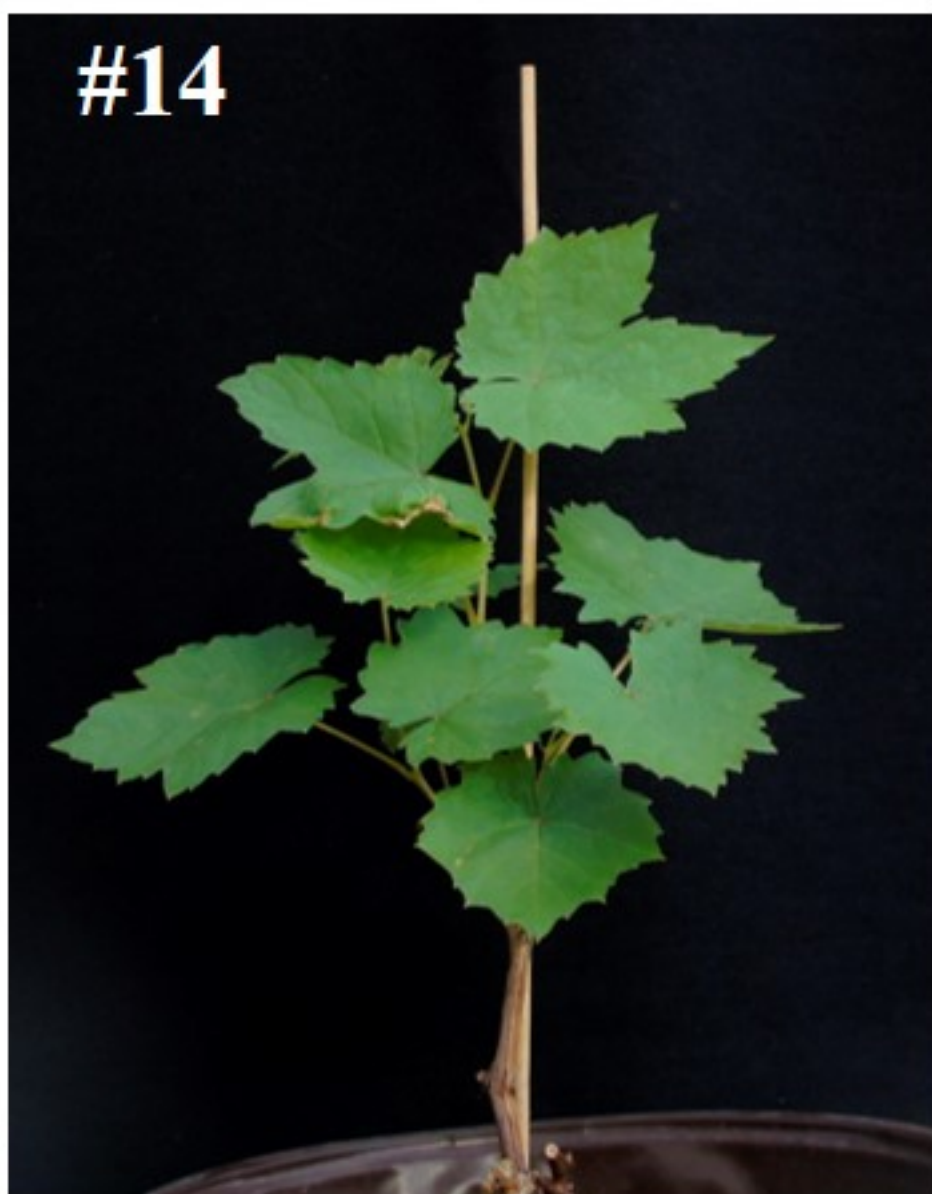

**#15**

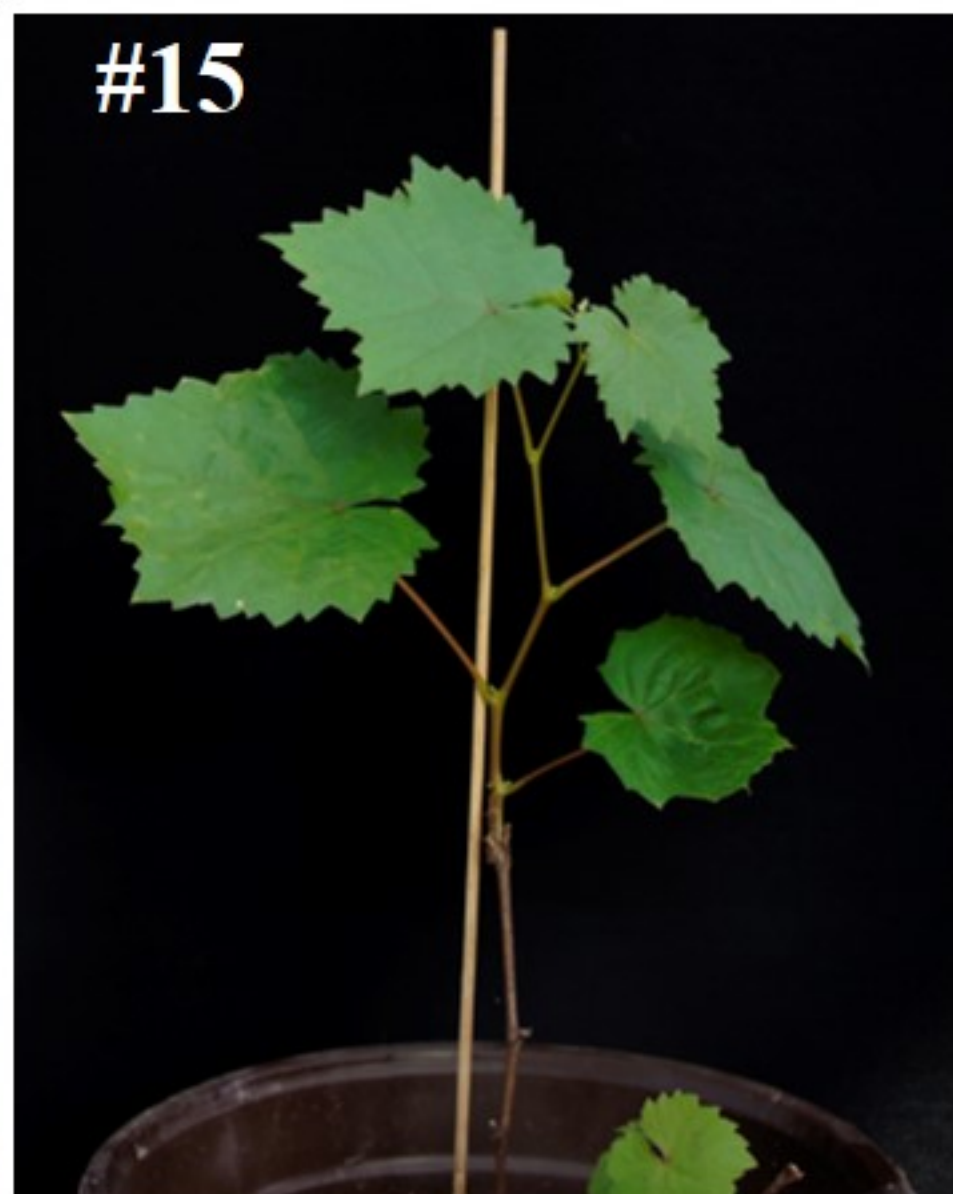

**#20**

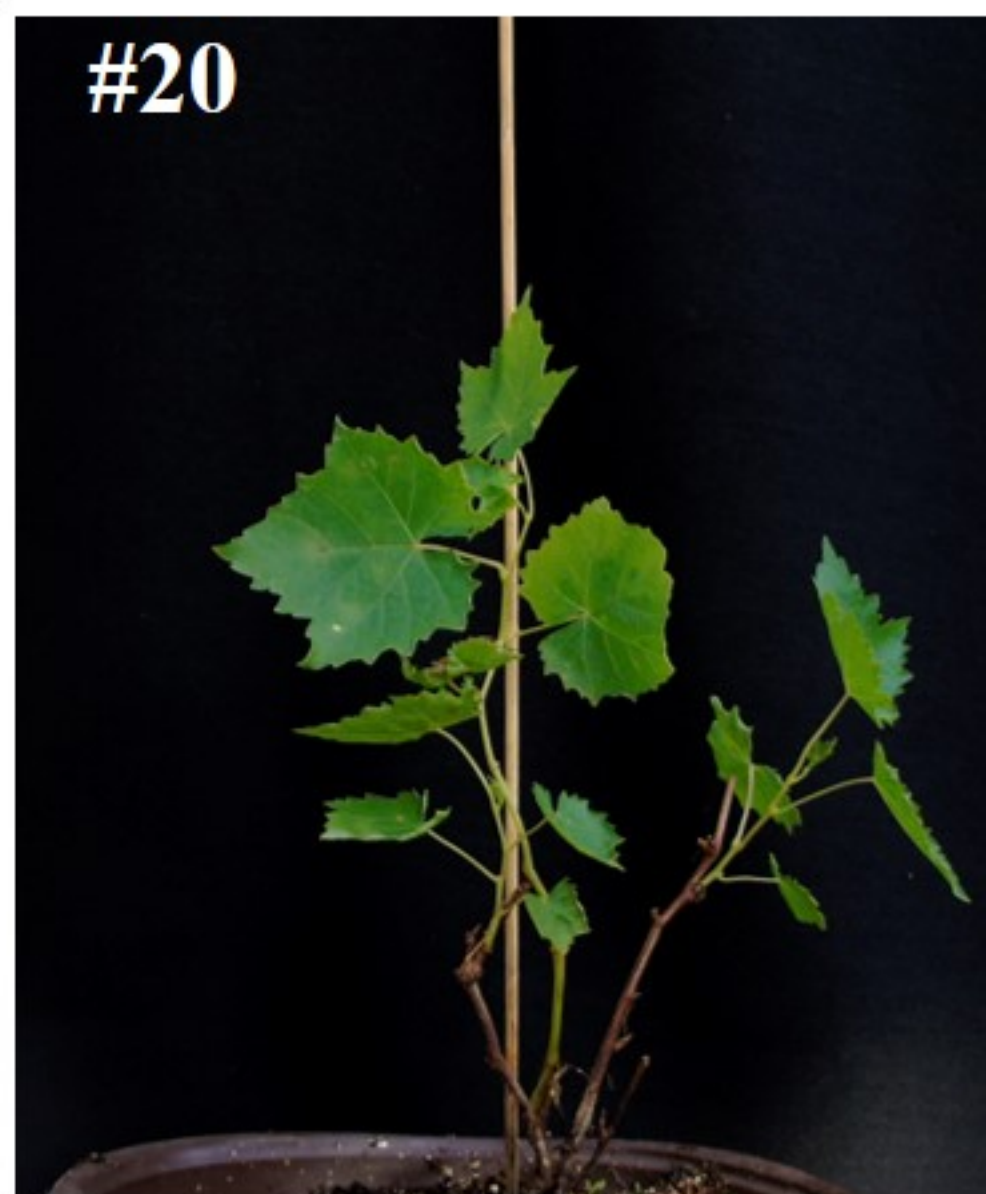

**#32**

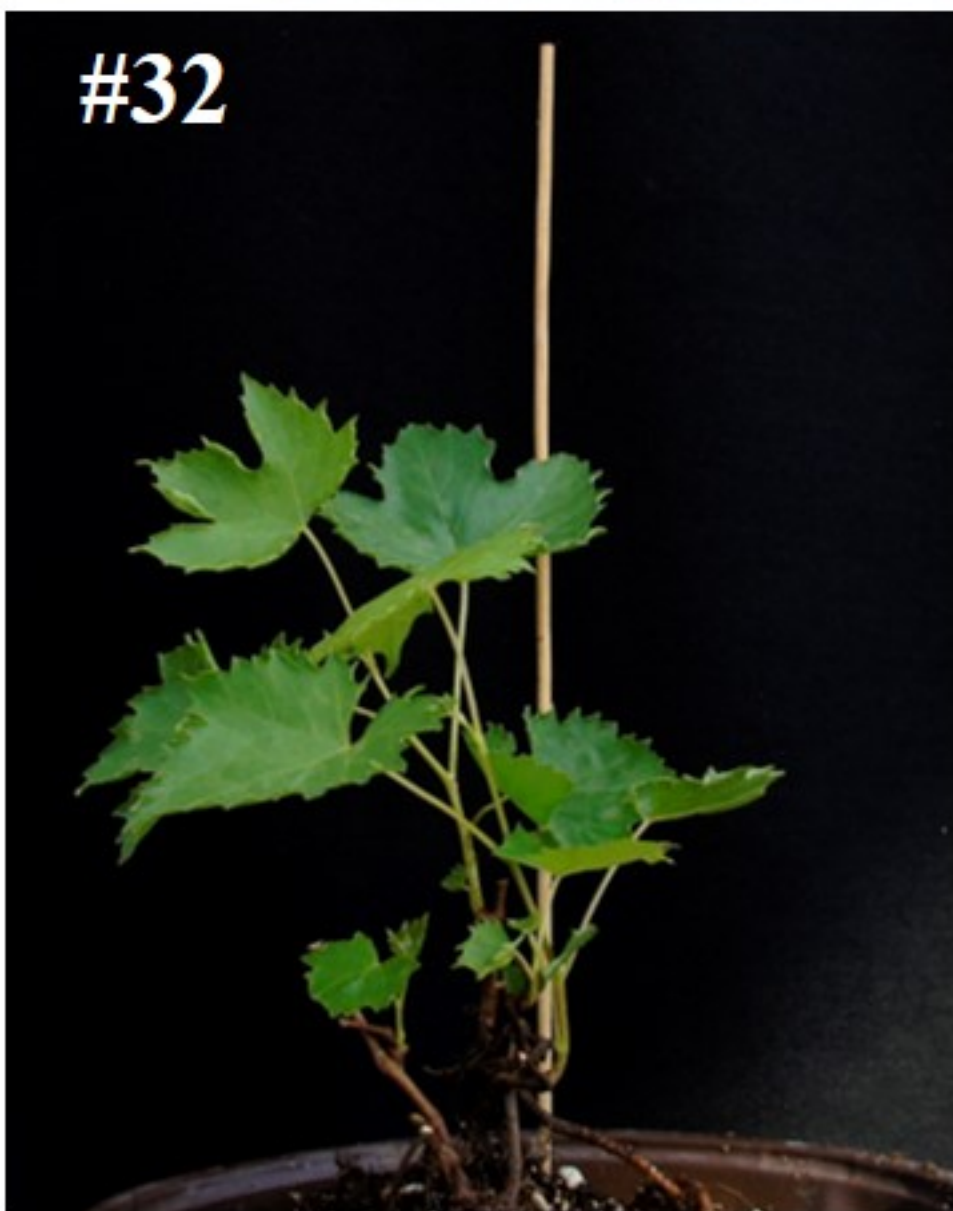

**#33**

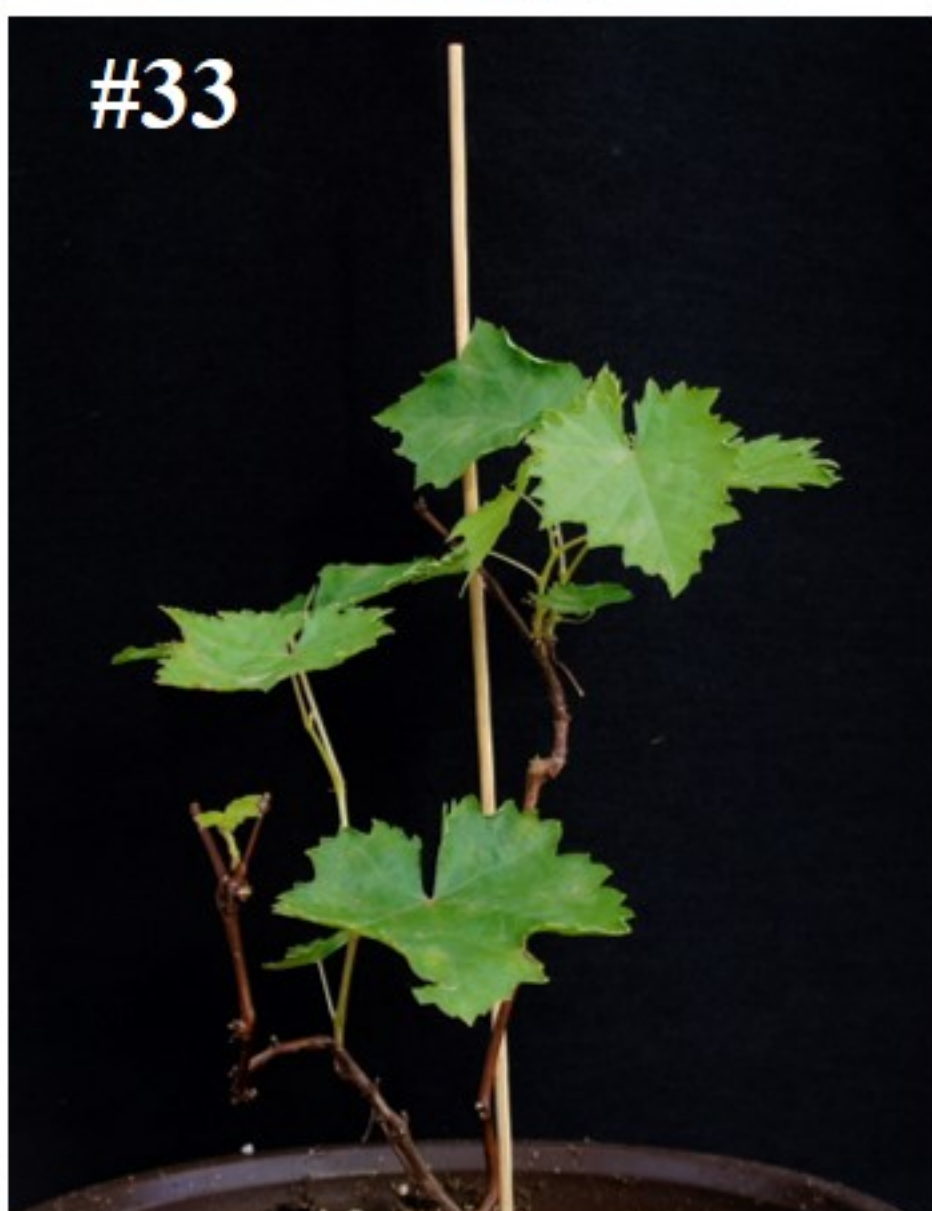

**#35**

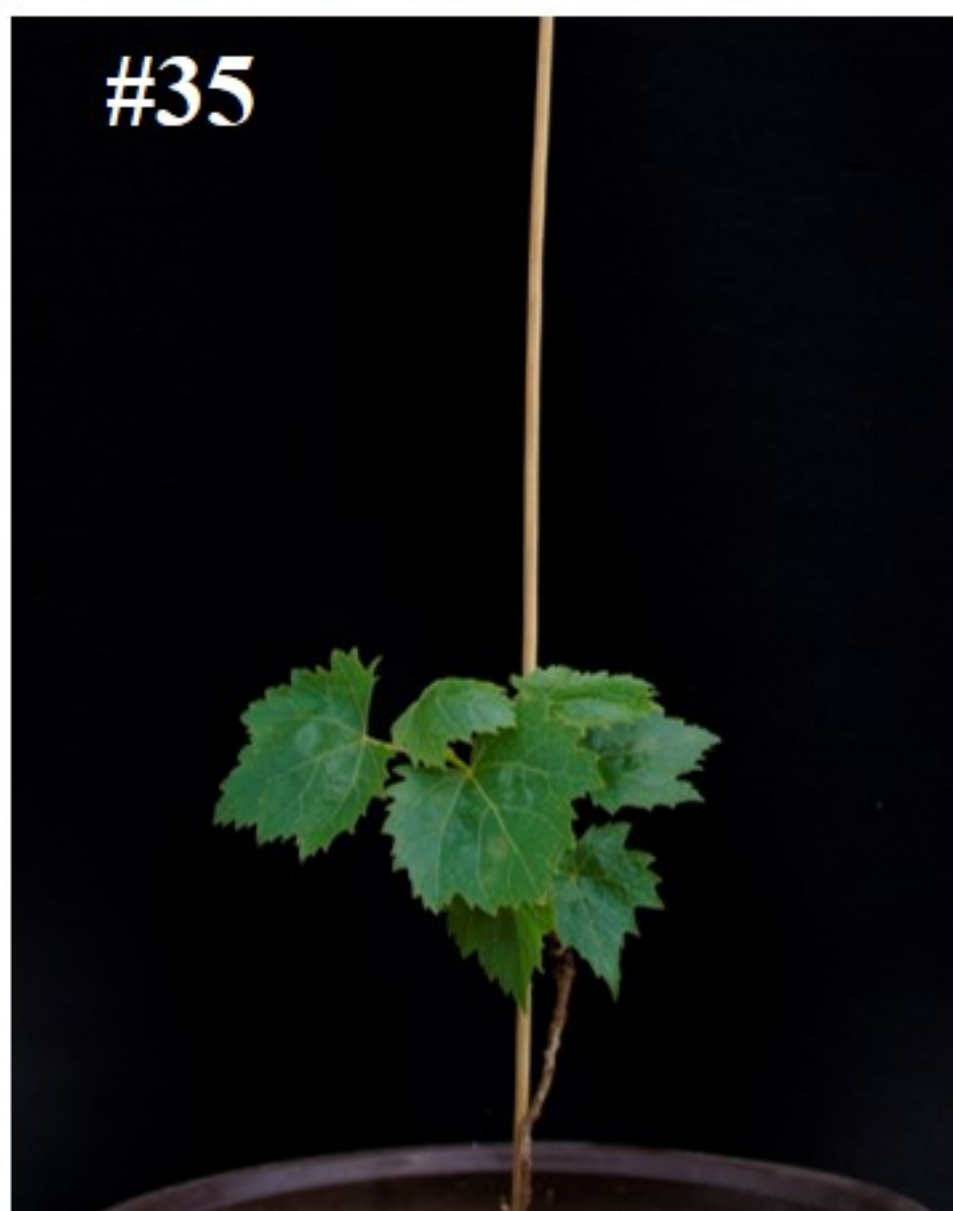

**#36**

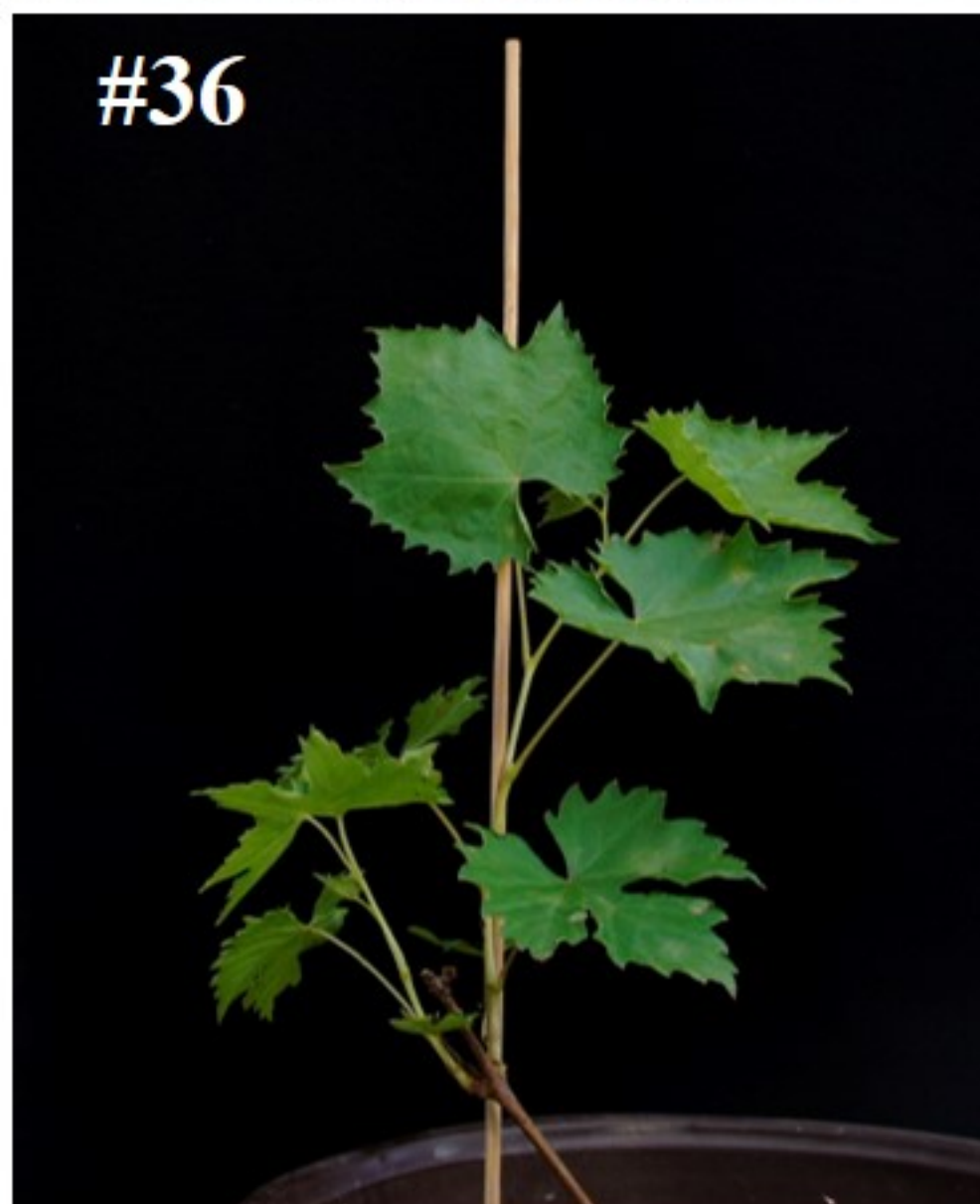

Supplement: Web_Material_uhab064 [file web_material_uhab064.zip › Fig. S2.pdf]

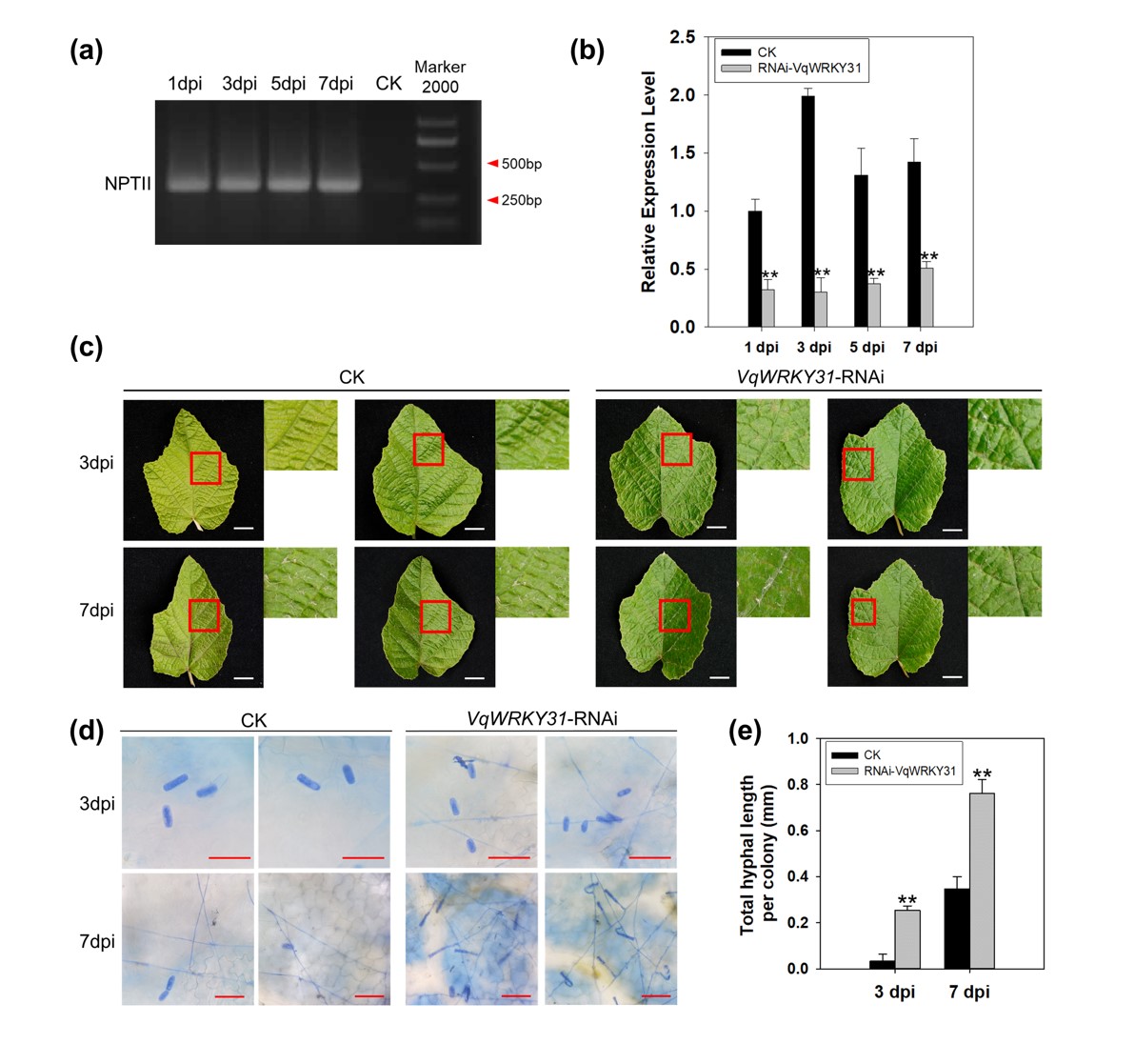

Supplement: Web_Material_uhab064 [file web_material_uhab064.zip › Fig. S3.jpg]

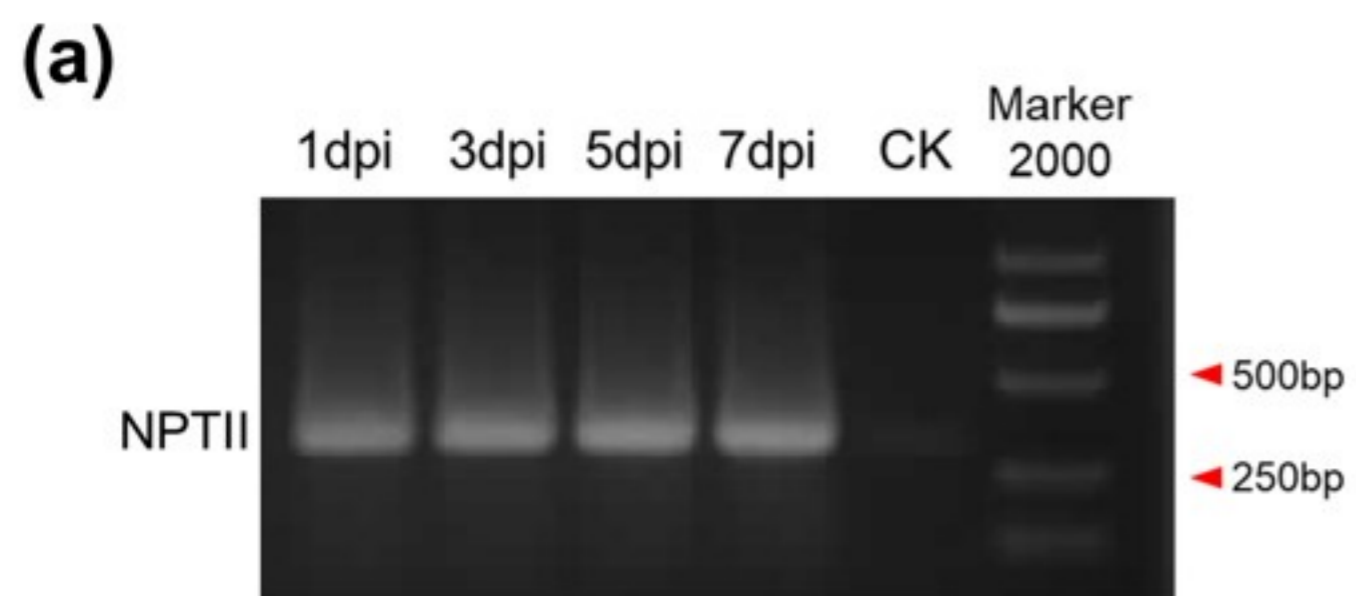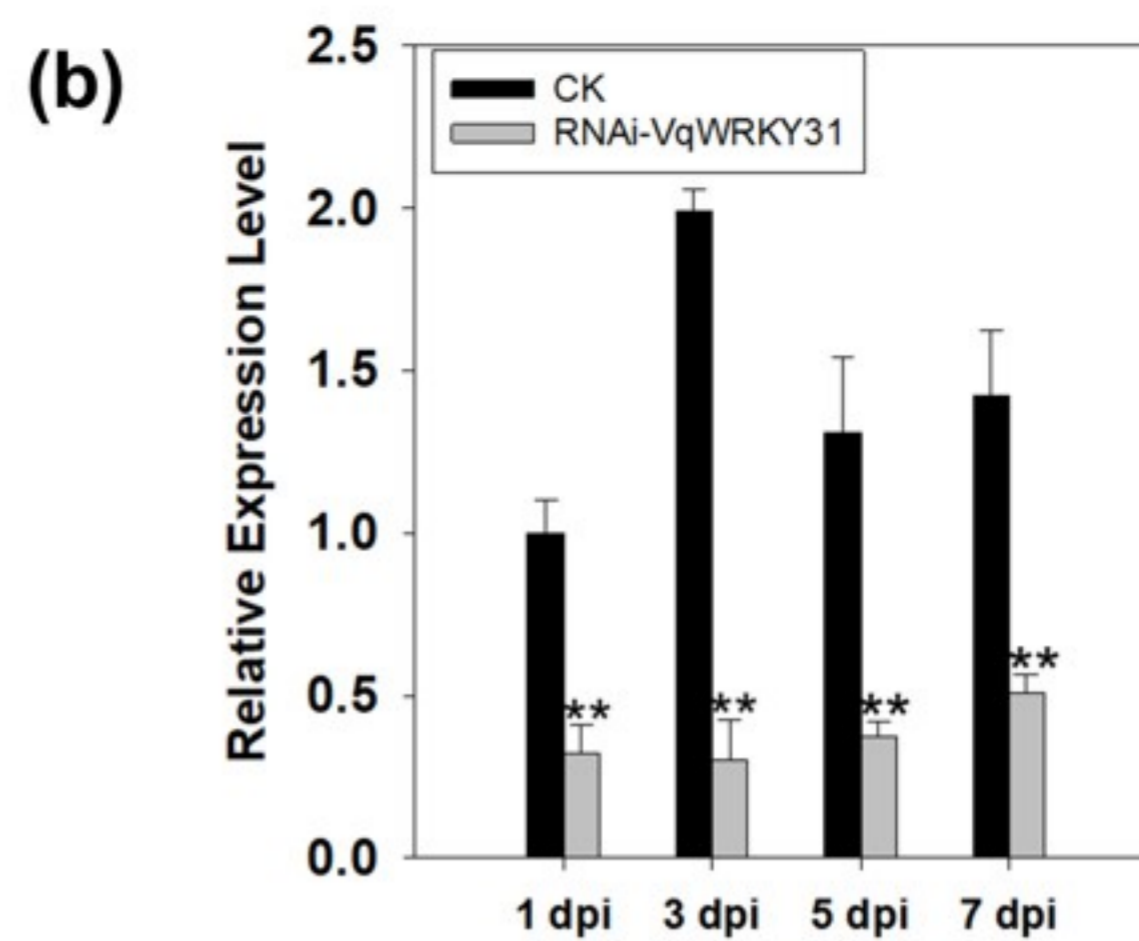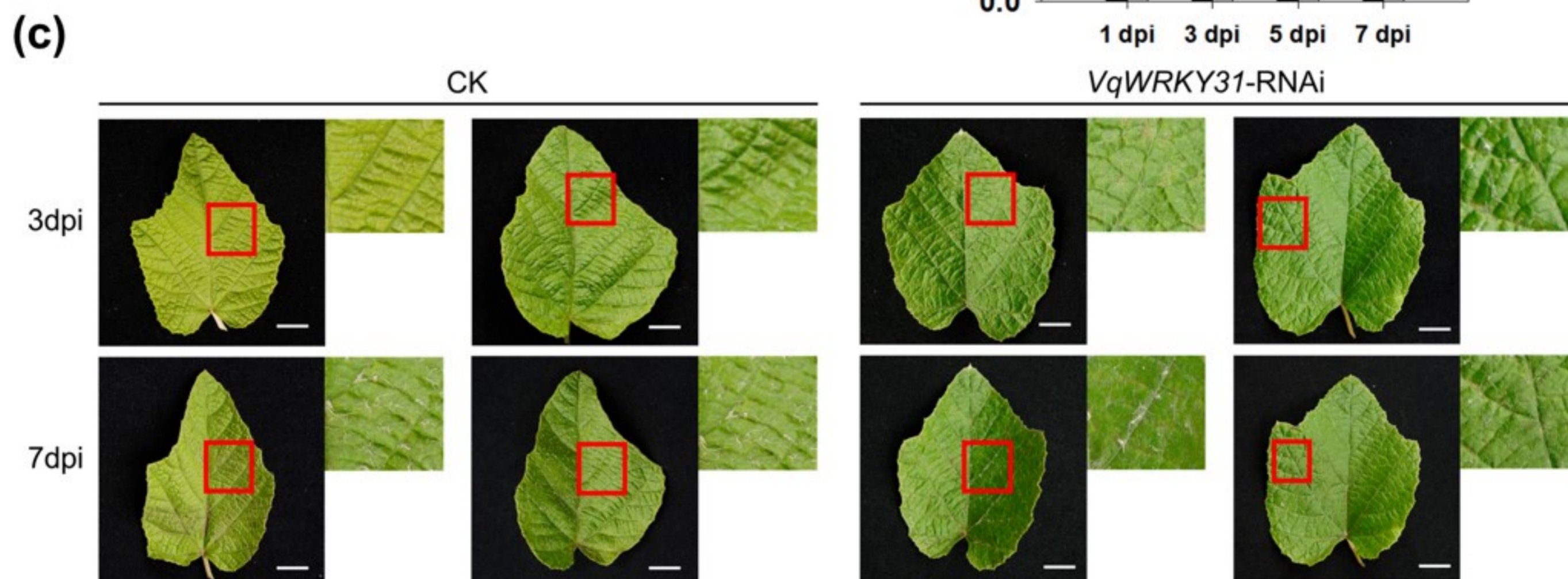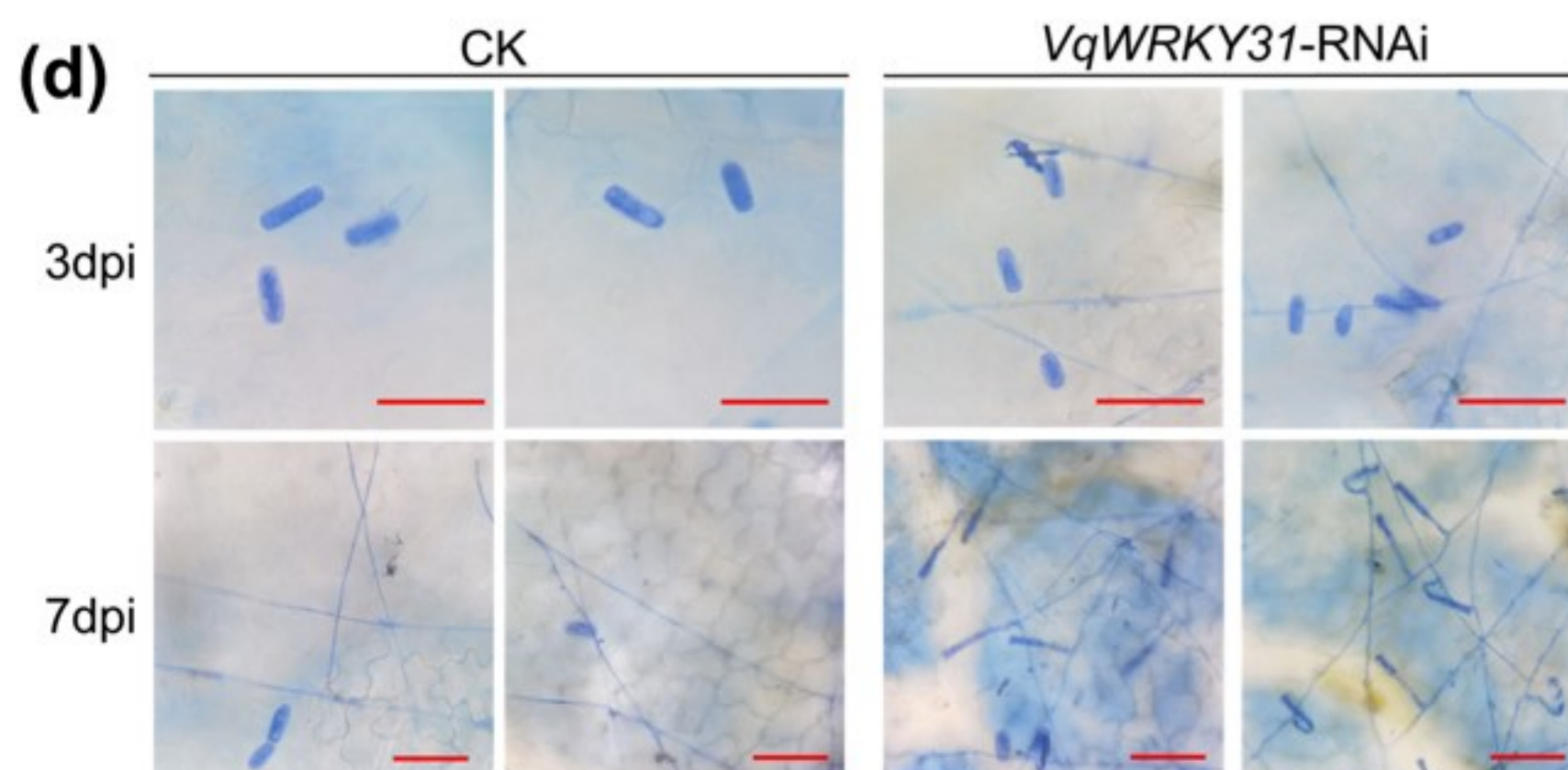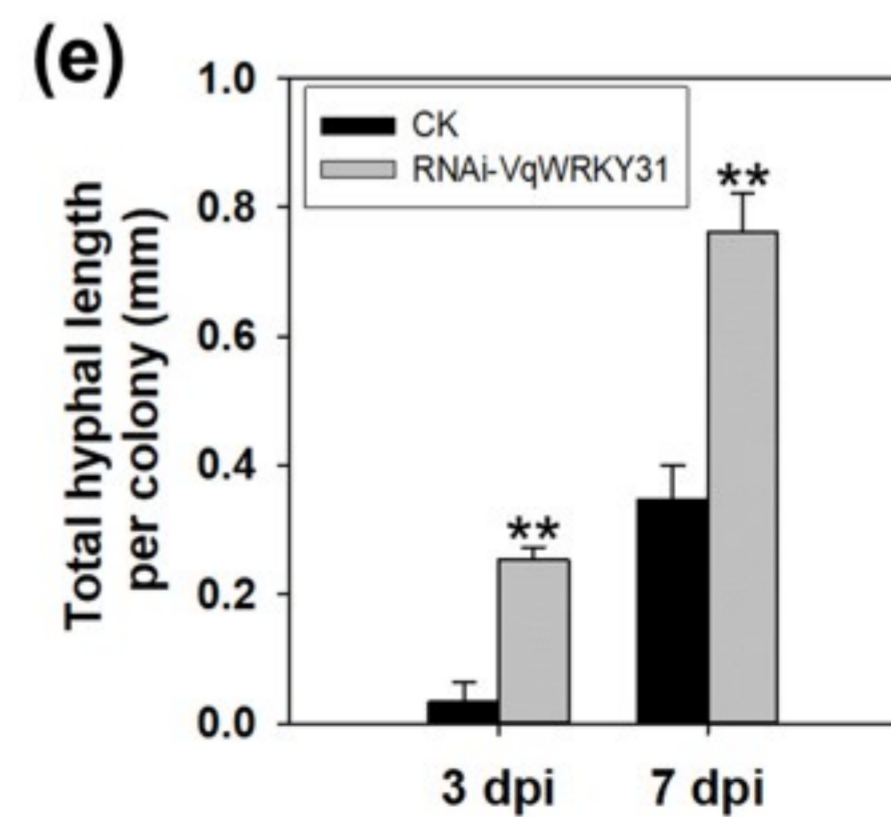

Supplement: Web_Material_uhab064 [file web_material_uhab064.zip › Fig. S3.pdf]

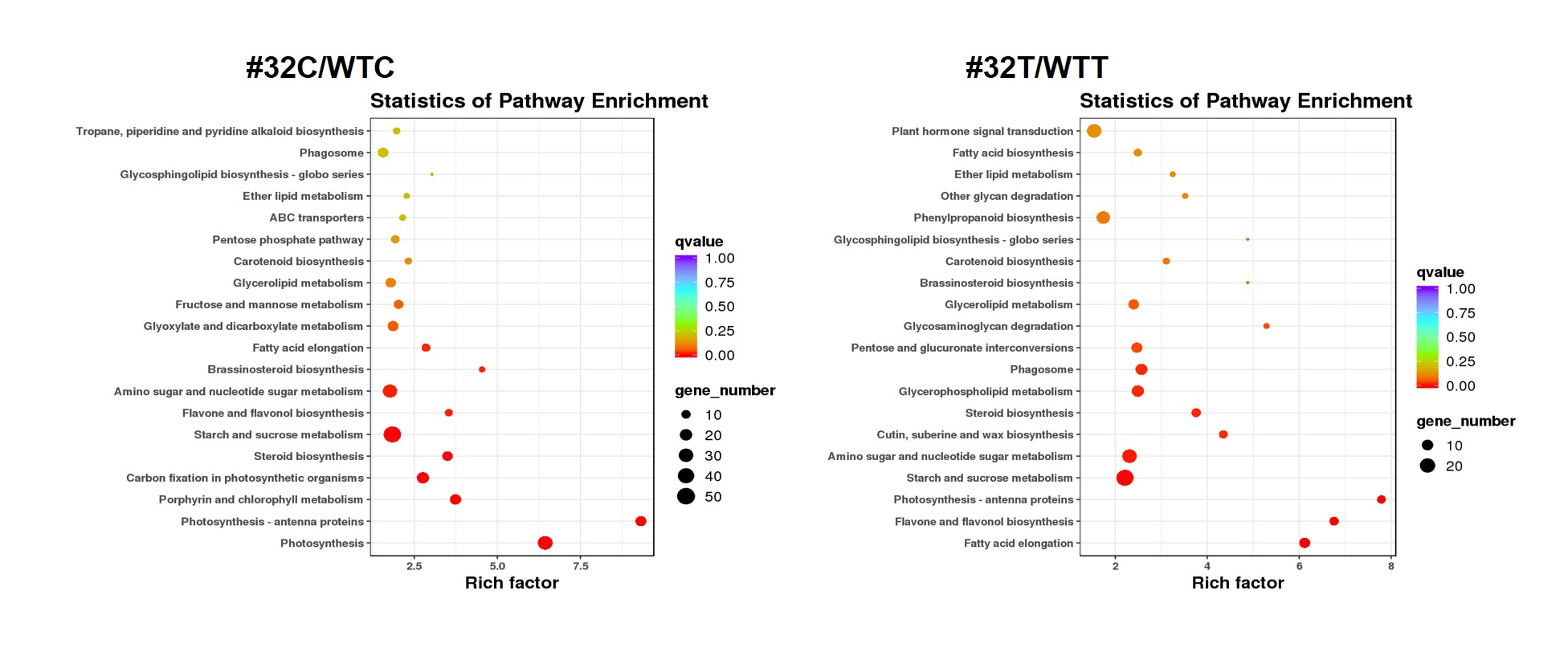

Supplement: Web_Material_uhab064 [file web_material_uhab064.zip › Fig. S4.jpg]

# #32C/WTC

## Statistics of Pathway Enrichment

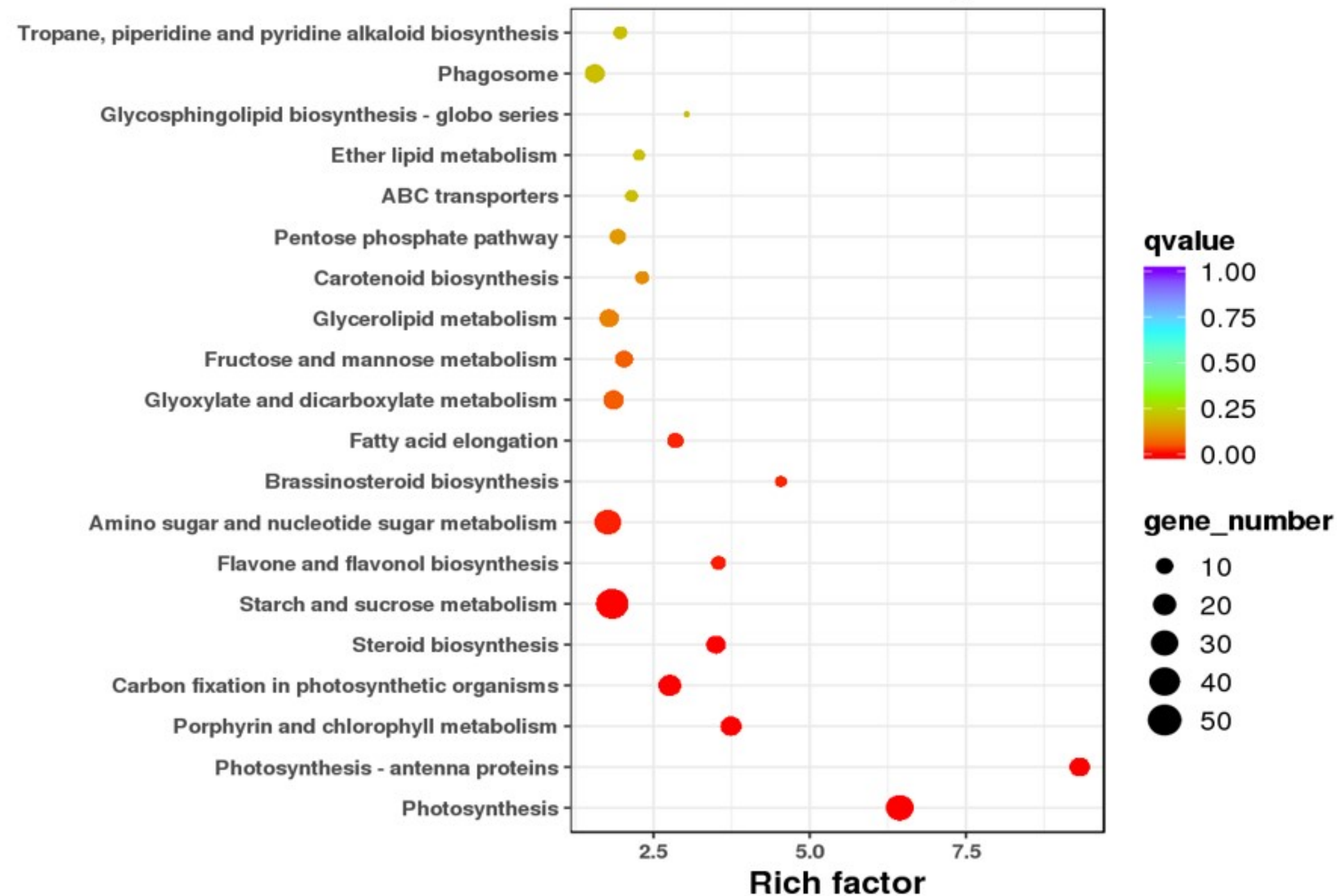

# #32T/WTT

## Statistics of Pathway Enrichment

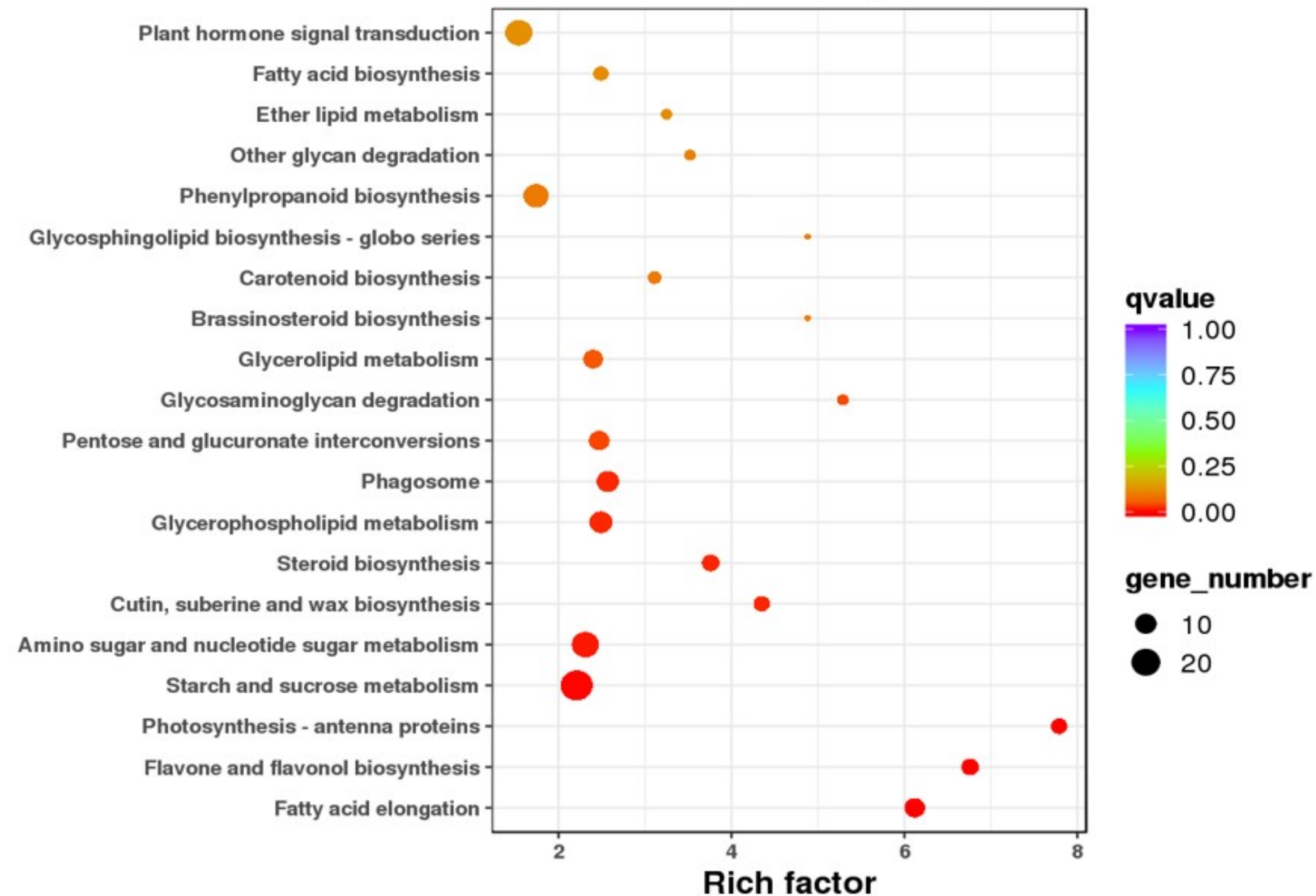

Supplement: Web_Material_uhab064 [file web_material_uhab064.zip › Fig. S4.pdf]

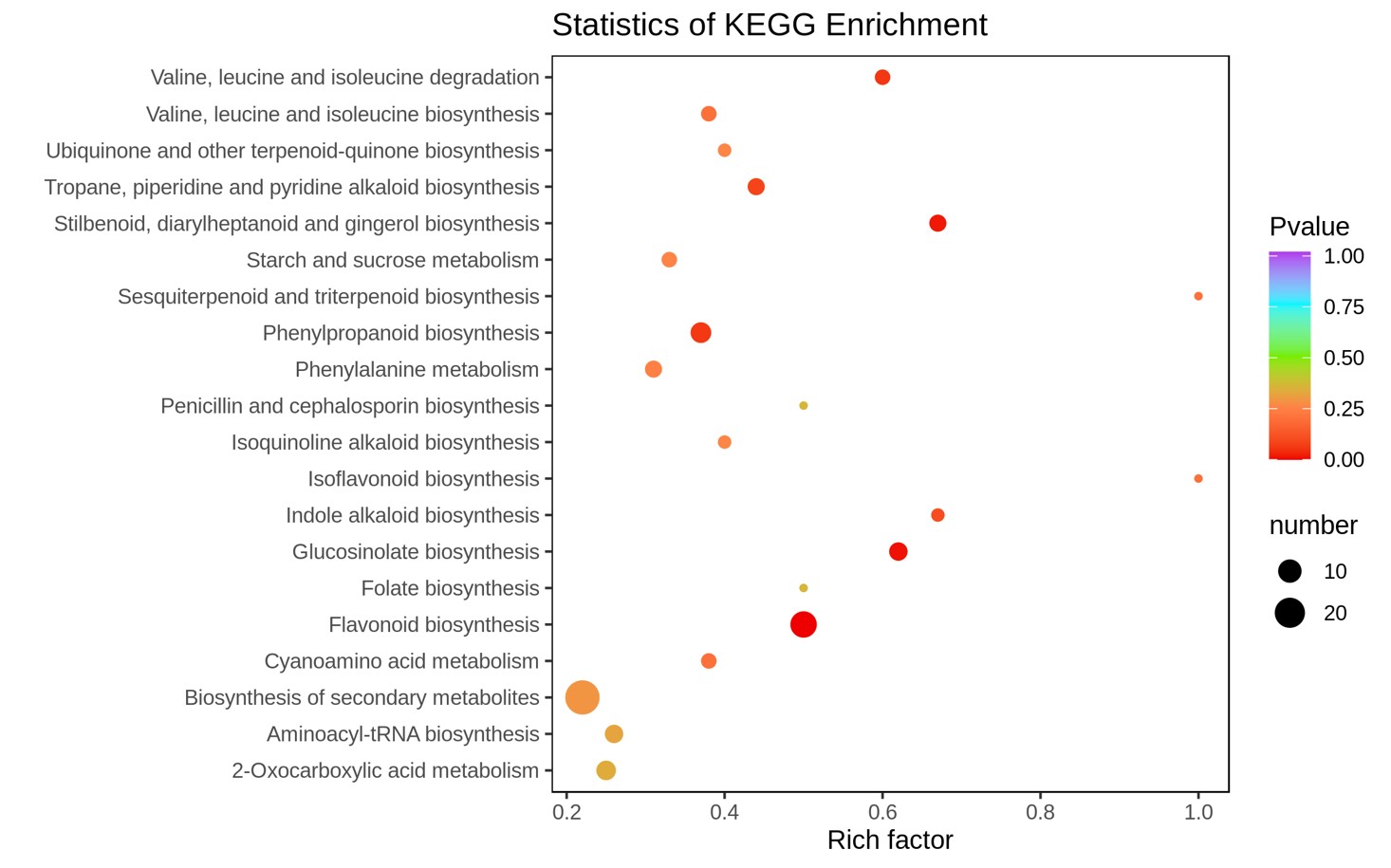

Supplement: Web_Material_uhab064 [file web_material_uhab064.zip › Fig. S5.jpg]

# Statistics of KEGG Enrichment

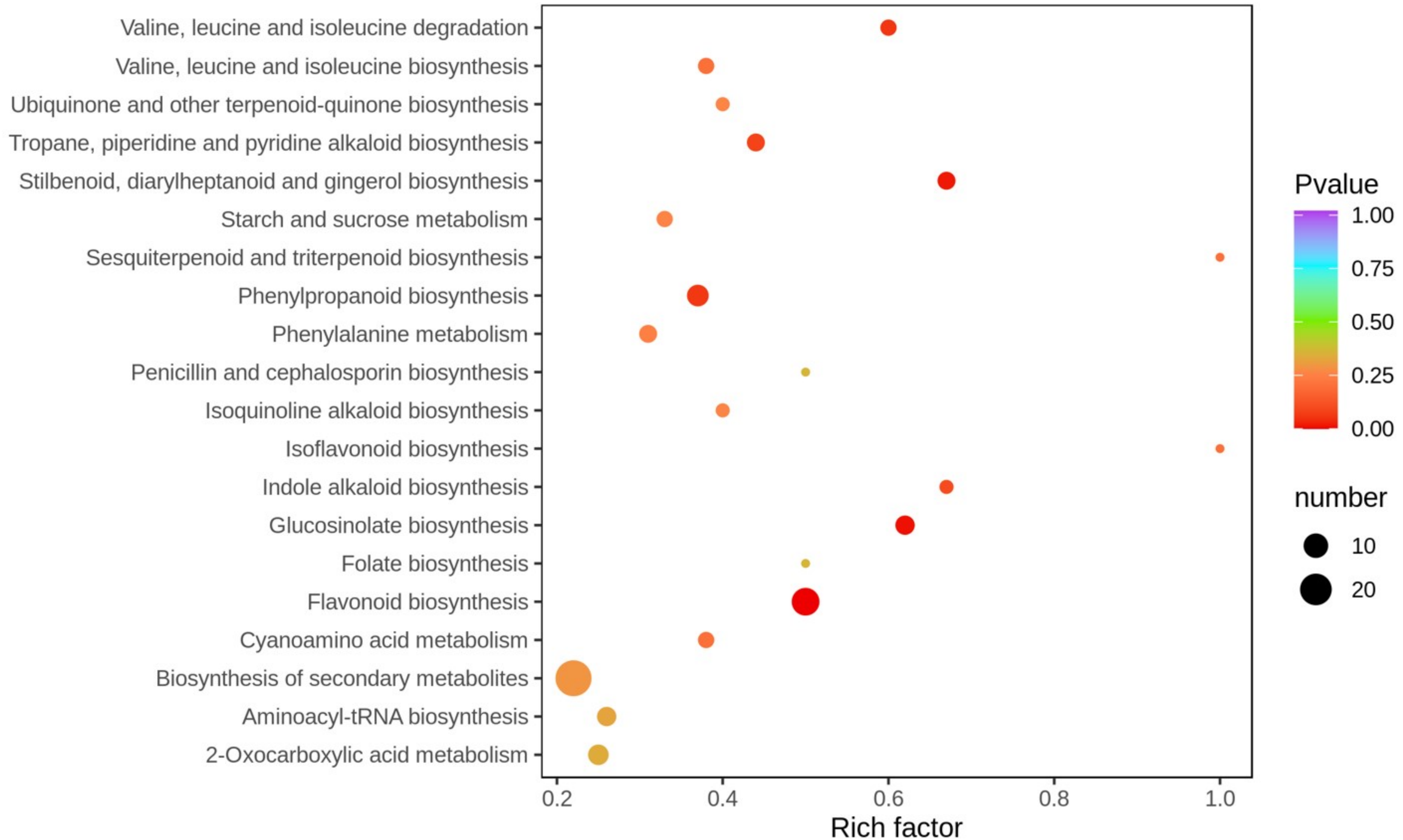

Supplement: Web_Material_uhab064 [file web_material_uhab064.zip › Fig. S5.pdf]

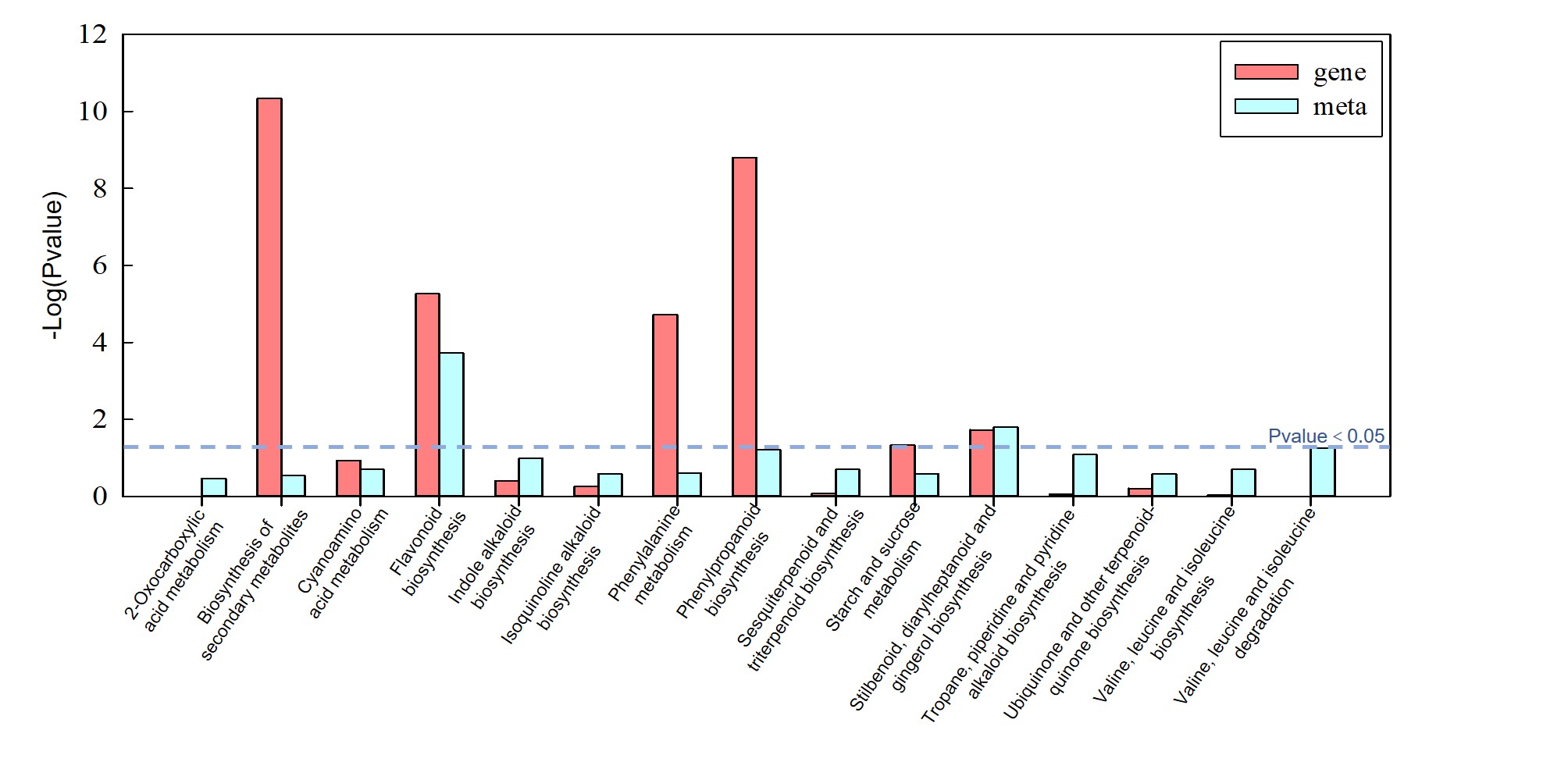

Supplement: Web_Material_uhab064 [file web_material_uhab064.zip › Fig. S6.jpg]

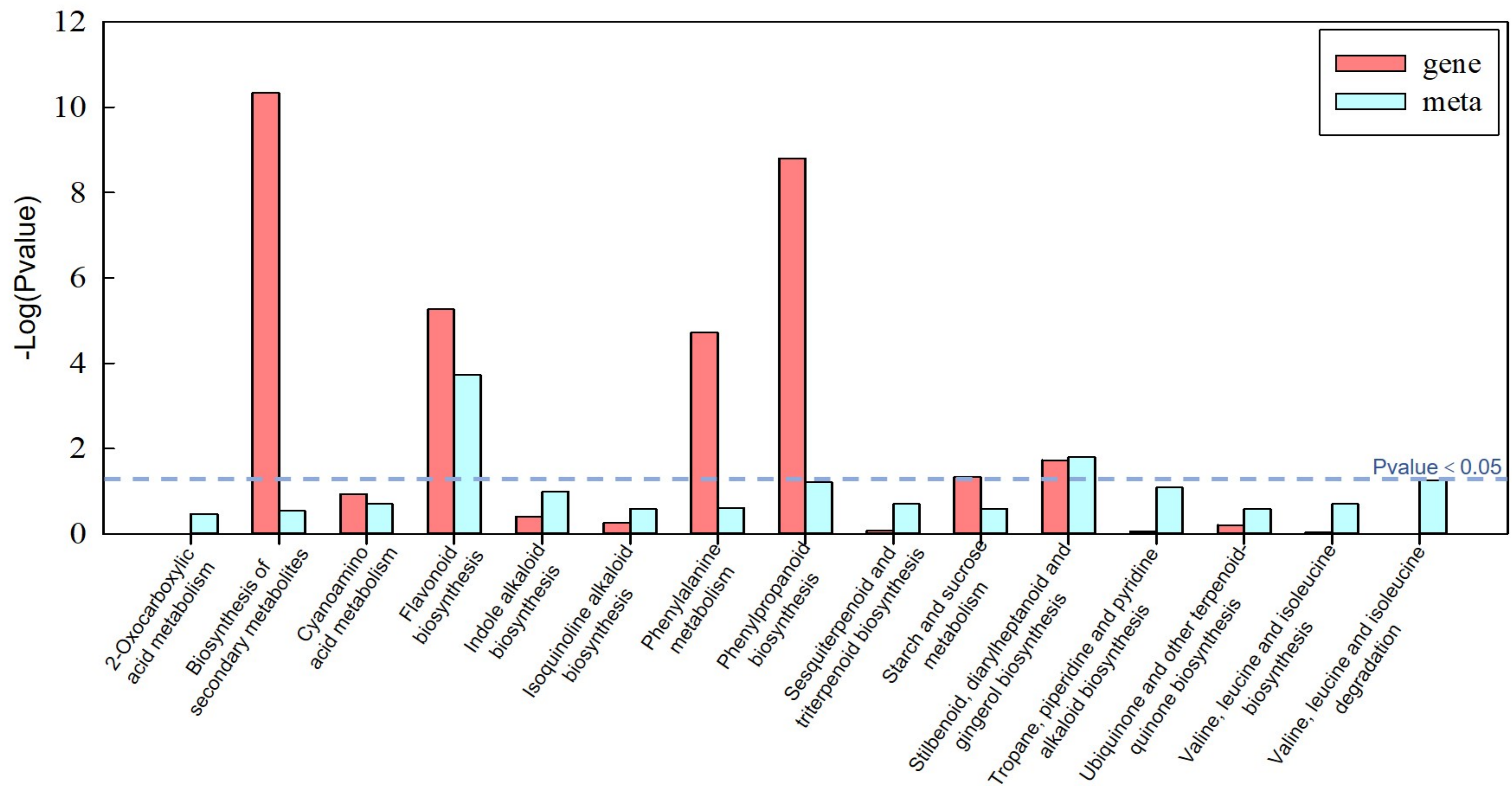

Supplement: Web_Material_uhab064 [file web_material_uhab064.zip › Fig. S6.pdf]
